# Mapping a network for tics in Tourette syndrome using causal lesions and structural alterations

**DOI:** 10.1093/braincomms/fcad105

**Published:** 2023-04-04

**Authors:** Jade-Jocelyne Zouki, Elizabeth G Ellis, Jordan Morrison-Ham, Phoebe Thomson, Aaron Jesuthasan, Bassam Al-Fatly, Juho Joutsa, Timothy J Silk, Daniel T Corp

**Affiliations:** Centre for Social and Early Emotional Development and School of Psychology, Deakin University, Geelong VIC 3220, Australia; Centre for Social and Early Emotional Development and School of Psychology, Deakin University, Geelong VIC 3220, Australia; Centre for Social and Early Emotional Development and School of Psychology, Deakin University, Geelong VIC 3220, Australia; Department of Paediatrics, The University of Melbourne, Melbourne VIC 3010, Australia; Developmental Imaging, Murdoch Children’s Research Institute, Melbourne VIC 3052, Australia; Autism Center, Child Mind Institute, New York NY 10022, USA; Neurology Department, Charing Cross Hospital, Imperial College Healthcare NHS Trust, London W6 8RF, UK; Department of Neurology with Experimental Neurology, Charité—Universitätsmedizin Berlin, corporate member of Freie Universität Berlin and Humboldt-Universität zu Berlin, Charitéplatz 1, 10117 Berlin, Germany; Turku Brain and Mind Center, Clinical Neurosciences, University of Turku, Turku, FI-20014, Finland; Turku PET Centre, Neurocenter, Turku University Hospital, Turku, FI-20520, Finland; Centre for Social and Early Emotional Development and School of Psychology, Deakin University, Geelong VIC 3220, Australia; Developmental Imaging, Murdoch Children’s Research Institute, Melbourne VIC 3052, Australia; Centre for Social and Early Emotional Development and School of Psychology, Deakin University, Geelong VIC 3220, Australia; Center for Brain Circuit Therapeutics, Department of Neurology, Psychiatry, and Radiology, Brigham and Women’s Hospital, Harvard Medical School, Boston, MA 02215, USA

**Keywords:** Tourette syndrome, tic disorders, functional connectivity, lesion network mapping, coordinate-based network mapping

## Abstract

Tics are sudden stereotyped movements or vocalizations. Cases of lesion-induced tics are invaluable, allowing for causal links between symptoms and brain structures. While a lesion network for tics has recently been identified, the degree to which this network translates to Tourette syndrome has not been fully elucidated. This is important given that patients with Tourette syndrome make up a large portion of tic cases; therefore, existing and future treatments should apply to these patients. The aim of this study was to first localize a causal network for tics from lesion-induced cases and then refine and validate this network in patients with Tourette syndrome. We independently performed ‘lesion network mapping’ using a large normative functional connectome (*n* = 1000) to isolate a brain network commonly connected to lesions causing tics (*n* = 19) identified through a systematic search. The specificity of this network to tics was assessed through comparison to lesions causing other movement disorders. Using structural brain coordinates from prior neuroimaging studies (*n* = 7), we then derived a neural network for Tourette syndrome. This was done using standard anatomical likelihood estimation meta-analysis and a novel method termed ‘coordinate network mapping’, which uses the same coordinates, yet maps their connectivity using the aforementioned functional connectome. Conjunction analysis was used to refine the network for lesion-induced tics to Tourette syndrome by identifying regions common to both lesion and structural networks. We then tested whether connectivity from this common network is abnormal in a separate resting-state functional connectivity MRI data set from idiopathic Tourette syndrome patients (*n* = 21) and healthy controls (*n* = 25). Results showed that lesions causing tics were distributed throughout the brain; however, consistent with a recent study, these were part of a common network with predominant basal ganglia connectivity. Using conjunction analysis, coordinate network mapping findings refined the lesion network to the posterior putamen, caudate nucleus, globus pallidus externus (positive connectivity) and precuneus (negative connectivity). Functional connectivity from this positive network to frontal and cingulate regions was abnormal in patients with idiopathic Tourette syndrome. These findings identify a network derived from lesion-induced and idiopathic data, providing insight into the pathophysiology of tics in Tourette syndrome. Connectivity to our cortical cluster in the precuneus offers an exciting opportunity for non-invasive brain stimulation protocols.

## Teaser

Zouki *et al*. map connectivity from lesions and structural alterations, identifying a common tic network relevant to Tourette syndrome, encompassing the basal ganglia and precuneus. Connectivity from the basal ganglia hub is abnormal in Tourette syndrome patients, validating this network. The precuneus hub offers opportunities for non-invasive brain stimulation protocols.

## Graphical Abstract

**Graphical Abstract:**
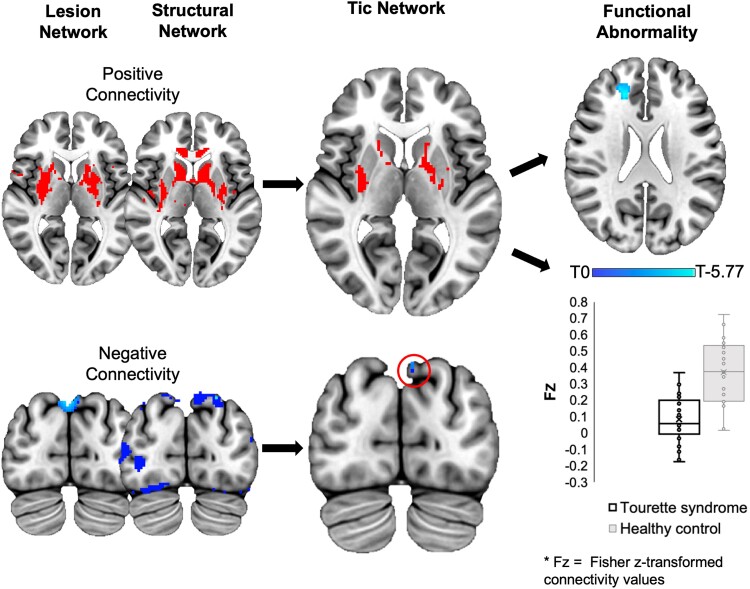

See Ganos and Horn (https://doi.org/10.1093/braincomms/fcad141) for a scientific commentary on this article.

## Introduction

Tics are defined by sudden stereotyped movements or vocalizations often resembling voluntary behaviour but with excessive repetition.^1,2^ Motor and vocal tics are commonly preceded by a premonitory urge or sensation,^3,4^ together comprising the hallmark symptoms of the most well-characterized idiopathic tic disorder, Tourette syndrome, which has a global prevalence of ∼1%.^5,6^ Tic pathophysiology is not yet fully understood, and a growing body of research continues to investigate the neural mechanisms underlying their presentation.^7^

Neuroimaging and neuropathology studies provide strong evidence for basal ganglia involvement in tic expression.^8,9^ Indeed, abnormal development of circuits that link the striatum and frontal cortex is proposed to play a key role in the generation and maintenance of tics.^9,10^ Alterations in the associative and sensorimotor regions of the striatum and globus pallidus are also implicated in the disinhibited behaviours defining tics.^11,12^ Beyond the basal ganglia, structural and functional neuroimaging studies in patients with Tourette syndrome demonstrate abnormalities within the sensorimotor network,^13–16^ prefrontal cortex,^17–22^ parietal operculum,^23,24^ insula^23–26^ and thalamus.^19,27^ These results suggest that tics may emerge from the dysfunction of multiple cortical and subcortical nodes within a distributed brain network.^22,28^ However, there is often a lack of reproducibility across these neuroimaging findings; therefore, the precise regions of this tic network are yet to be defined.^10,14,27,29^

While tics are primarily idiopathic, occasionally, they can occur secondary to a focal brain lesion.^30^ These cases are invaluable as they allow for causal links to be drawn between symptoms and brain structures.^31–34^ However, lesions causing tics occur in multiple regions, leaving localization unclear.^35–37^ ‘Lesion network mapping’ (LNM) is a novel neuroimaging technique introduced to address this difficulty by mapping lesions to brain networks rather than anatomical locations.^31^ The LNM technique leverages a large external data set (*n* = 1000) of resting-state functional connectivity MRI (rs-fcMRI) scans in healthy volunteers (termed ‘connectome’) to isolate the brain regions that are commonly connected to lesions causing a given neurological symptom.^38,39^ Recently, Ganos *et al*.^40^ used LNM to show that lesions causing tics were commonly connected to a network involving the insular cortices, cingulate gyrus, striatum, globus pallidus internus (GPi), thalami and cerebellum, supporting the involvement of a distributed brain network in tic generation.

Although these findings localized regions associated with lesion-induced tics, the degree to which this network is also abnormal in idiopathic patients with Tourette syndrome has not been fully elucidated. The application of LNM in tics may also implicate structures unrelated to Tourette syndrome and those strongly connected to brain lesions in general. ‘Coordinate network mapping’ (CNM) is a technique similar to LNM; however, instead of mapping brain lesions, CNM maps coordinates of structural differences between patients and healthy controls from published neuroimaging studies.^41^ To date, this technique has successfully been applied to Alzheimer’s disease, Parkinson’s disease dementia, migraine and depression.^41–44^ The advantage of this technique is that networks can be derived from idiopathic populations, who comprise the majority of cases in most disorders, rather than patients with lesion-induced symptoms. However, using CNM alone lacks causal inference.

The aim of this study was to first independently perform LNM to localize a tic network from lesion-induced cases and then to use CNM in Tourette syndrome studies to test whether there are brain structures that are both causally linked to tics and abnormal in patients with Tourette syndrome. Finally, to validate our findings in individuals with Tourette syndrome, we assessed whether connectivity from the identified network hubs is abnormal in an independent sample of rs-fcMRI data from Tourette syndrome patients compared with healthy controls.

## Materials and methods

### Case and coordinate selection

For the LNM analysis, cases of brain lesions causing motor and/or vocal tics were identified through searches of PubMed and Embase databases in March 2021, using a combination of synonyms for the following terms: ‘Tourette*’, tics, lesion, stroke, infarct, ischaemia, haemorrhage, tumour, plaque and brain injury (see Supplementary material for exact search syntax). Inclusion criteria were (i) neurological assessments documenting tics that were believed to be induced by a focal brain lesion and (ii) an image demonstrating the brain lesion in which clear lesion borders could be identified. Exclusion criteria were (i) large or diffuse lesions involving both grey and white matter, spanning multiple sections of functionally heterogeneous brain tissue^45^ (see next paragraph); (ii) large cysts distorting brain structure;^33^ (iii) lesions of the CNS but outside of the brain, such as meningiomas;^32^ and (iv) reports of tic improvement post brain lesioning. One reviewer (J.-J.Z.) screened the titles and abstracts using EndNote (version X9) and Rayyan software^46^ before assessing full-text reports in EndNote. All included cases were reviewed for eligibility by the senior authors (J.J./T.S./D.C.). Reference lists of included reports were assessed for potential cases missed in the initial search.

A similar analysis of lesion-induced tics was published during data analysis.^40^ All aspects of our LNM analysis, including the systematic search, lesion tracing and connectivity analyses were performed independently of this study, yet using similar and previously validated LNM methods.^32,45^ However, based on a recent review of the LNM technique,^45^ we applied more stringent criteria for the inclusion of lesions. Specifically, we excluded large or diffuse lesions involving both grey and white matter, spanning multiple sections of functionally heterogeneous brain tissue. These lesions were excluded for several reasons. First, LNM uses rs-fcMRI, which is limited in how it can account for disrupted white matter contributing to symptoms.^45^ Second, lesion network maps are derived by using lesions as ‘seeds’ to extract the average blood-oxygen-level-dependent (BOLD) signal within the lesion volume. Therefore, averaging the BOLD signal over many different regions of grey and white matter likely results in less precision and strength of lesion connectivity.^45^ Third, the location, structure and boundaries of diffuse lesions are often difficult to determine and therefore trace onto a standard brain atlas.

After our analyses were complete, Ganos *et al*.^40^ shared their lesions for possible cases that were missed in the initial search and for analyses of network agreement (see below). Two additional cases were identified at this time, which were later excluded for not meeting our aforementioned criteria (i.e. large or diffuse lesion or unclear lesion boundaries). A further nine cases included by Ganos *et al*.,^40^ also identified in our search, were excluded for not meeting this same criteria. (See Supplementary Table 1 for exclusion reasons for each lesion.) We assessed spatial correlations between the lesions included in our analysis and that of Ganos *et al*.^40^ following validated methods^47^ (Supplementary material). Strong spatial correlations were observed between shared lesions (*n* = 11, *r* = 0.868), confirming similar lesion tracings. Comparison of lesions that were unique to our data set (*n* = 8) and that of Ganos *et al*.^40^ (*n* = 11) produced a spatial correlation of *r* = 0.344. Overall, strong spatial convergence was identified between the present and previously published LNM analyses, with network overlap in the insular cortices, cingulate gyrus, basal ganglia, thalamus and cerebellum (Supplementary Fig. 1).

For the structural coordinate mapping analyses, MEDLINE Complete and Embase databases were searched in March 2020, with an updated search in August 2021, for neuroimaging studies in patients with idiopathic Tourette syndrome. A combination of synonyms for the following terms was used: ‘Tourette* syndrome’, magnetic resonance imaging, voxel-based morphometry, single-photon emission computed tomography, positron emission tomography and atrophy (see Supplementary material for full search syntax). Inclusion of studies required (i) a neuroimaging technique to compare Tourette syndrome patients to healthy controls; (ii) reporting of whole brain analysis;^48^ and (iii) reporting of stereotaxic coordinates of significant brain differences amongst patients. Two reviewers (J.-J.Z./J.M.-H.) independently screened the titles and abstracts of the search results using EndNote and Rayyan software,^46^ with full-text articles assessed for eligibility in EndNote. Disagreements regarding the inclusion of full-text articles were resolved by a senior author (D.C.). Reference lists of included articles were assessed for possible studies missed in the initial search.

### Localization of lesion-induced tics

Each lesion from the included cases was hand-traced by authors J.-J.Z. and D.C. onto a standard 2 × 2 × 2 MNI152 brain atlas using FSLeyes^49^ (version 0.34.2). The accuracy of all lesion tracings was verified by a consultant neurologist (J.J.). Next, we identified the network of brain regions functionally connected to each lesion location. A standard seed-based analysis technique was used following validated methods described elsewhere,^31,32^ leveraging the aforementioned normative functional connectome.^50,51^ Functional connectivity maps were thresholded at a *t*-value of ± 7 [corresponding to whole brain family-wise error (FWE) corrected *P* < 10^−6^] to create a binarized map of regions functionally connected to each lesion location.^32^ Binarized maps from each lesion were overlaid, separately for positive and negative connectivity, resulting in a network map for lesion-induced tics. Following visual inspection,^40^ the network map was restricted to include voxels connected to ≥ 16/19 lesions. This threshold was chosen to identify structures connected to the maximum number of lesions (higher thresholds retained minimal to no voxels; see Supplementary Fig. 2 for results at different thresholds). These voxels were defined as ‘sensitive’ to lesion-induced tics.

To identify voxels that are both sensitive and specific to this network, we compared the connectivity of lesions causing tics to a control data set comprised of lesions causing other movement disorders. To prevent bias, lesions were sourced from all the movement disorder data sets within our lab repository. Functional connectivity *t*-maps derived from the included lesions were compared to those from cervical dystonia, *n* = 25;^32^ parkinsonism, *n* = 29;^33^ and Holmes tremor, *n* = 36,^34^ using a general linear model (GLM) with 1000 permutations. All analyses were conducted using Statistical Parametric Mapping (SPM12; http://www.fil.ion.ucl.ac.uk/spm/software/spm12/).^52^ This approach identified voxels that are significantly more (or less) connected to lesions causing tics compared to control lesions.^32^ A threshold-free cluster enhancement method was used to correct for multiple comparisons, with corrected *P* < 0.05 considered significant.^34,53^ Finally, we restricted these specificity maps to voxels sensitive to lesions causing tics, defining regions both sensitive and specific to lesion-induced tics.

### Localization of structural alterations in Tourette syndrome

We then investigated networks from structural brain coordinates in Tourette syndrome using two methods. First, we performed anatomical likelihood estimation (ALE) meta-analysis using GingerALE (version 3.0.2; http://brainmap.org/ale/). The analysis tested for regions of consistent significant structural differences, that is, higher or lower regional grey and white matter volume in Tourette syndrome patients relative to controls. Exploratory analyses were conducted using two contrasts. The first contrast examined regions showing higher volume in patients relative to controls, with the second testing for areas where volume was lower amongst patients compared to controls. The analyses were conducted with a cluster-forming threshold of *P* < 0.001 (uncorrected), 1000 permutations, and corrected for multiple comparisons using a cluster-level interference threshold of FWE *P* < 0.05^54^ (see Supplementary material for further detail). Further exploratory analyses are presented in the Supplementary material and Supplementary Table 2.

**Figure 1:**
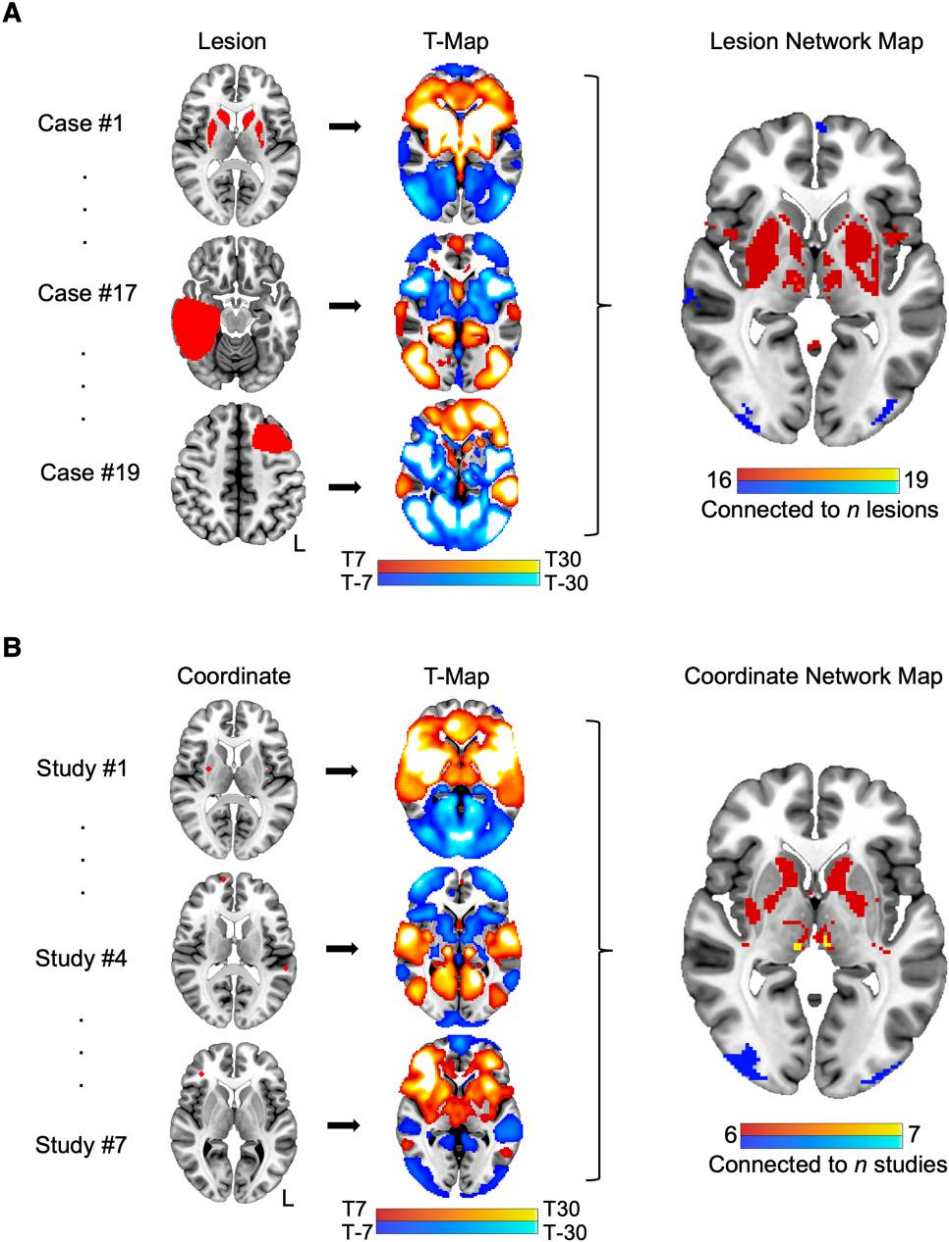
**Lesion and coordinate network mapping techniques.** (**A**) Lesions causing tics were hand-traced onto a standard brain atlas. The network of brain regions functionally connected to each lesion were identified using a normative functional connectome (*n* = 1000). The resultant functional connectivity maps were thresholded, binarized and overlaid to identify voxels connected to ≥ 16/19 lesions. (**B**) Spherical seeds were generated at each coordinate of structural brain abnormality in Tourette syndrome from the included studies and pooled to create a single combined seed for each study. Following the same approach described above, combined study seeds were used as inputs for seed-based analysis. Finally, thresholded and binarized maps were overlaid to identify voxels connected to ≥ 6/7 combined study seeds. Lesion and coordinate network maps (rightmost panel) are shown at *z* = 0 to demonstrate the methods; see Fig. 4A and D for results. Case and study numbers correspond to those listed in Supplementary Tables 5 and 6, respectively.

As ALE meta-analysis does not assess the connectivity of coordinates, we then performed CNM to assess whether this method would result in higher convergence of brain regions across studies. CNM was conducted according to validated methods described elsewhere.^41–43^ Spherical seeds (4 mm) were generated at each reported coordinate of significant structural differences between patients and controls from the included studies and pooled to create a single combined seed for each study. Next, we identified the network of brain regions functionally connected to each study’s combined seed. This procedure involves the same steps described above for the LNM analysis; however, combined study seeds were used as inputs.^41–43^ Functional connectivity maps were thresholded at a *t*-value of ± 7 to create a binarized map of regions functionally connected to each study’s combined seed.^42,43^ Finally, binarized maps from each study were overlaid, separately for positive and negative connectivity, to represent a network map for Tourette syndrome. As per LNM, the coordinate network map was restricted to include voxels connected to the maximum number of combined study seeds (≥ 6/7; higher threshold retained minimal voxels). (See Supplementary Fig. 3 for results provided at different thresholds.) Figure 1A and B show a summary of the LNM and CNM techniques.

### Mapping a network for tics in Tourette syndrome

As per the aim of the study, we used these LNM and CNM networks to define a tic network relevant to patients with Tourette syndrome. Specifically, we sought to perform a conjunction analysis to identify brain structures from the ‘sensitive and specific’ LNM network that are also common to the networks derived from structural coordinate mapping using ALE meta-analysis and CNM. The ALE meta-analysis failed to identify any significant consistent structural differences between patients and controls at threshold (FWE *P* < 0.05). Exploratory analysis only identified a small cluster contributed to by two out of six studies, which showed no overlap with the lesion network. Accordingly, the conjunction analysis was performed using the LNM and CNM networks. All positive and negative connectivity analyses were performed separately.

### Network validation in Tourette syndrome patients

The positive and negative networks derived from the above conjunction analysis were used as regions of interest to compare functional connectivity between patients with Tourette syndrome and healthy controls.

Resting-state fcMRI data from patients with diagnosed Tourette syndrome and healthy controls were acquired from the Healthy Brain Network (HBN), an ongoing biobank comprising neuroimaging and phenotypic data from children and adolescents within the New York area. Data were collected at three imaging sites. Our criteria for the selection of scans included in the present analyses were (i) available subject-specific field maps and (ii) application of the same repetition time for all participant scans in order to combine rs-fcMRI data in the analyses. The HBN inclusion and exclusion criteria for the cohort and the imaging parameters used can be found at the databank website (http://fcon_1000.projects.nitrc.org/indi/cmi_healthy_brain_network/). Our motion limit for volume exclusion was set at framewise displacement > 0.5 mm.^55^ Following current guidelines,^56^ we included participants in the analyses if they had ≥ 4 min of uncensored data, corresponding to at least 300 volumes. After removing 2 patients and 2 controls with excessive motion, the sample consisted of 21 children and adolescents diagnosed with Tourette syndrome and 25 healthy controls. (See Supplementary Table 3 for demographic and clinical characteristics of the sample.) Consent was obtained in accordance with the Declaration of Helsinki. Legal guardians provided written informed consent, while participants assented.^57^ This study was approved by the Deakin University Research Ethics Committee (HREC #2021-424).

Data were pre-processed using fMRIprep (version 20.2.3; RRID:SCR_016216)^58,59^ and the Functional Connectivity (CONN) Toolbox (version 19.c; http://www.nitrc.org/projects/conn; RRID:SCR_009550).^60^ We applied conventional pre-processing steps, including discarding the first four volumes to allow for stabilization of the BOLD signal, estimation of motion parameters using MCFLIRT (FSL; version 5.0.9)^61^ and spatial normalization to MNI space. Automatic removal of motion artefacts was performed using independent component analysis (ICA-AROMA)^62^ after the removal of non–steady-state volumes and spatial smoothing (6-mm full-width half-maximum isotropic Gaussian kernel). Volumes with excessive motion and physiological artefactual effects (white matter, cerebrospinal fluid and their first-order derivatives) were removed during the denoising stage by including these as regressors. Scans were band-pass filtered between 0.008 and 0.09 Hz after regression to identify fluctuations in the BOLD signal.^63^

Standard seed-based analyses were used to compare functional connectivity from the seed regions between Tourette syndrome patients and controls. Time courses of the BOLD signal were extracted from the seed regions and correlated with all other brain voxels. The resultant connectivity maps were entered into GLMs for group comparison.

To assess whether connectivity from our conjunction networks was selectively abnormal in patients with Tourette syndrome, we replicated this seed-based analysis in the ‘sensitive and specific’ LNM networks identified in the same three aforementioned control data sets involving other movement disorders (cervical dystonia, parkinsonism and Holmes tremor).^32–34^ (See Supplementary Table 4 for further detail on these control networks.)

### Statistical analysis

All statistical comparisons between patients with Tourette syndrome and healthy controls were conducted using the CONN Toolbox^60^ and IBM SPSS Statistics^64^ (version 28.0.1.0). Differences in seed-based connectivity between groups were assessed using GLMs, controlling for age, sex and imaging site. The significance of the analyses was assessed using a height threshold of *P* < 0.001 (two-sided) and a FWE cluster-size correction of *P* < 0.05.^65^ In-scanner motion, based on mean framewise displacement, was compared between groups using a two-sample *t*-test (*P* < 0.05, two-sided).

## Results

### Cases of lesion-induced tics and structural alterations in Tourette syndrome

**Figure 2:**
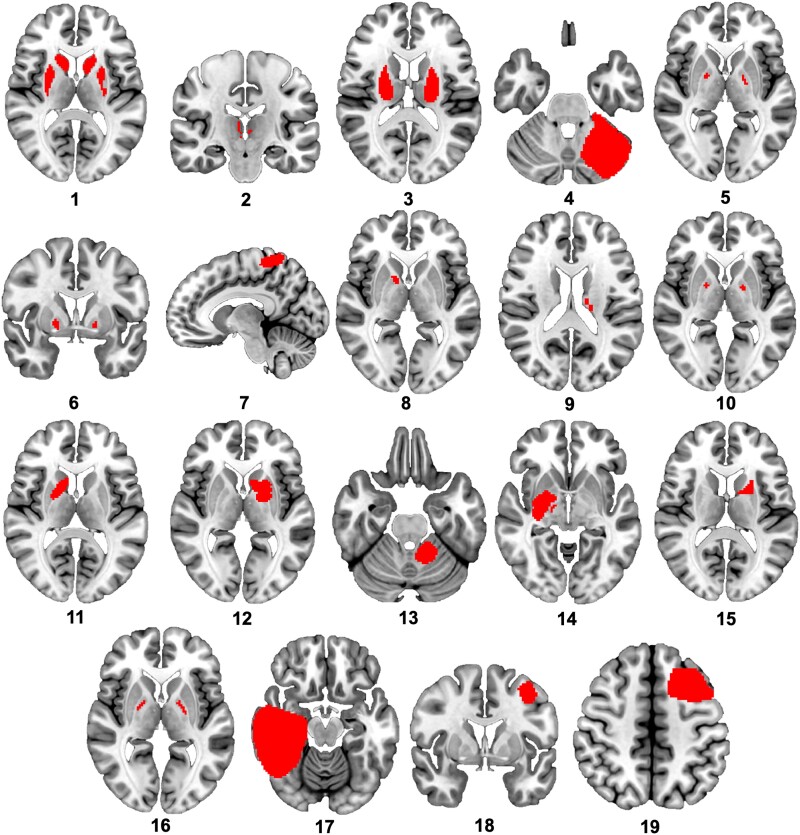
**Lesion locations causing tics**. A systematic literature search identified 19 cases of lesion-induced tics that included a focal lesion that could be drawn onto a standard atlas of the brain. Lesions were mostly found within the basal ganglia, yet also within other brain structures, such as the frontal, temporal and parietal lobes, thalamus, internal capsule, cerebellum and brainstem. Case numbers correspond to those in Supplementary Table 5, which provides further clinical detail for each case.

For the LNM analysis, titles and abstracts of 3529 studies were screened, with 19 cases of lesion-induced tics meeting inclusion criteria (see Supplementary Fig. 4 for the systematic search flowchart and Fig. 2 for individual lesion tracings). Of the included cases, the average age at tic onset was 27.1 years (± 20.7 *SD*, age range = 6–71 years; in two cases, age at tic onset was not reported). Most cases (*n* = 11) demonstrated co-occurring motor and vocal tics. Of the eight remaining cases, seven presented with isolated motor tics, while one demonstrated vocal tics exclusively. The presence of premonitory urge was reported in 7 cases, and suppressibility of tics was documented in 10 cases. Lesions were most commonly reported within the basal ganglia (putamen, caudate nucleus, globus pallidus; 13 cases) but were also present in multiple other brain regions, including the frontal, temporal and parietal lobes, thalamus, internal capsule, cerebellum and brainstem. Lesion aetiology varied across cases, including stroke, infection and traumatic brain injury (Supplementary Table 5). (For detailed case-specific information regarding the manifestation of tics, latency between brain lesioning and tic onset and the presence of co-occurring movement disorders and neuropsychiatric symptoms, see Supplementary Table 5.)

**Figure 3:**
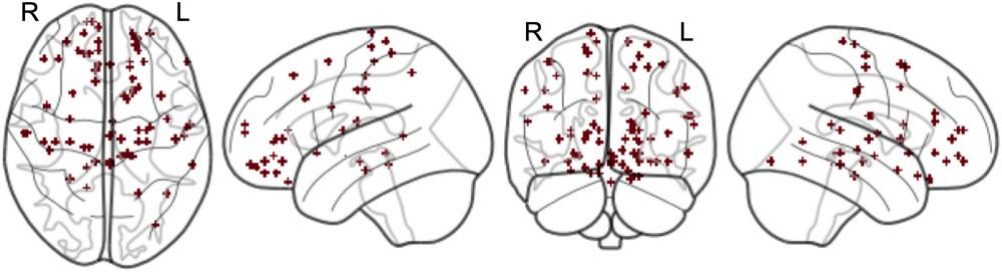
**Distribution of structural brain alterations in Tourette syndrome**. Coordinates (*n* = 77) sourced from seven studies reporting higher or lower volume in idiopathic Tourette syndrome patients compared to healthy controls, displayed as 4-mm spheres on a standard brain atlas. These coordinates were distributed throughout multiple cortical and subcortical regions.

A total of 567 studies were assessed for eligibility for the ALE and CNM analyses (see Supplementary Fig. 5 for the systematic search flowchart). Seven articles reporting significant structural differences in patients with Tourette syndrome relative to healthy controls met inclusion criteria. All studies used voxel-based morphometry to measure regional grey and white matter volume across the whole brain. Coordinates of brain alterations were reported in multiple cortical and subcortical structures (Fig. 3). (See Supplementary Table 6 for a summary of the neuroimaging findings and the demographic and clinical sample characteristics of the included studies. For coordinates used in the ALE and CNM analyses, see Supplementary Table 7.)

### Localization of lesion-induced tics

**Figure 4:**
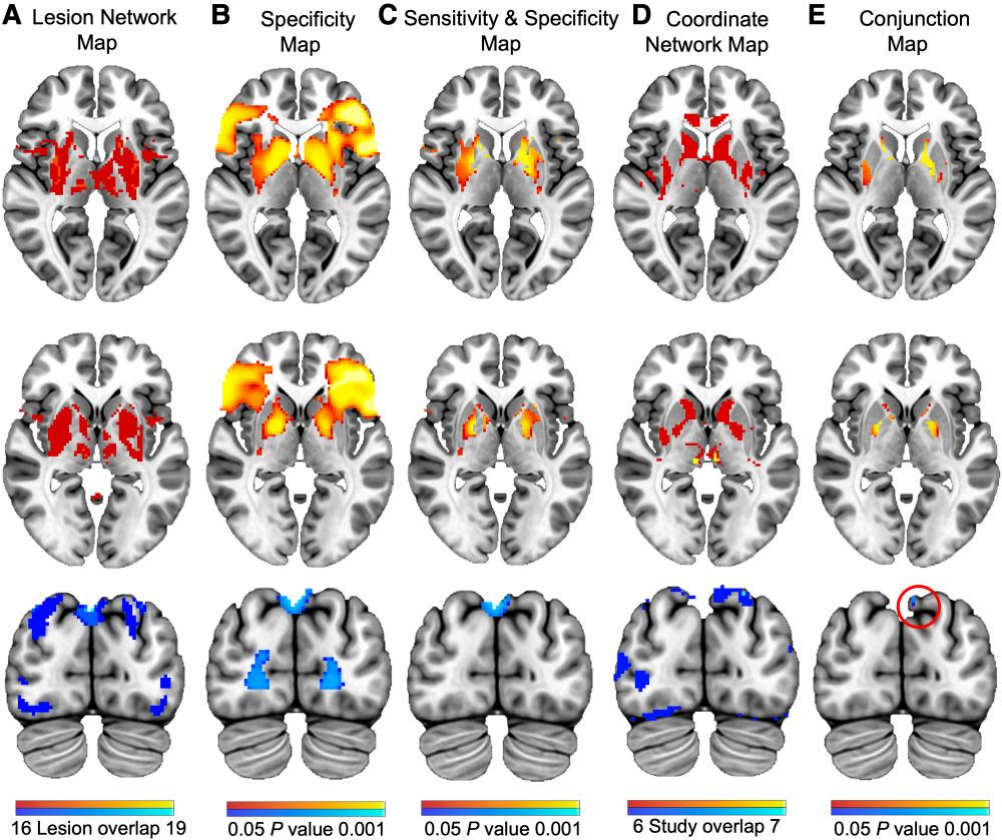
**Mapping a network for tics in Tourette syndrome.** (**A**) LNM findings showing regions positively (top heat bar) or negatively (bottom heat bar) correlated to lesions causing tics. (**B**) Positive and negative connections specific to lesions causing tics compared to those causing other movement disorders (cervical dystonia, parkinsonism, Holmes tremor) identified using a GLM with 1000 permutations. (**C**) Voxels both sensitive and specific to lesions causing tics. (**D**) CNM findings showing regions positively or negatively correlated to structural alterations in idiopathic Tourette syndrome. (**E**) Conjunction analysis showing a final network involving voxels common to the ‘sensitive and specific’ lesion (Fig. 4C) and coordinate (Fig. 4D) networks. From *top* to *bottom*: this conjunction network for tics involved the posterior putamen, caudate nucleus, GPe (*z* = 5, −1) and precuneus (*y* = −81.5).

Despite conducting a wider systematic search, applying more stringent criteria for the inclusion of lesions and independently performing LNM from Ganos *et al*.,^40^ the analyses localized similar brain structures (Supplementary Fig. 1). Lesions were predominantly connected (positively correlated) to the bilateral basal ganglia, as well as the insular cortices, cingulate gyrus, thalamus, midbrain and cerebellum. Of note, the present LNM identified novel negative connectivity, with all 19 lesions connected to the bilateral precuneus (Fig. 4A). Some smaller clusters showing positive and negative connectivity were also identified (Supplementary Figs. 6 and 7).

Voxels specific to lesions causing tics compared to the control movement disorders were mainly located within the bilateral basal ganglia, insular cortices, cingulate gyrus (positively correlated) and precuneus (negatively correlated; Fig. 4B). (See Supplementary Figs. 8 and 9 for all identified voxels.) Voxels within the bilateral basal ganglia, cingulate gyrus (positively correlated) and precuneus (negatively correlated) were both sensitive and specific to lesions causing tics (Fig. 4C).

### Localization of structural alterations in Tourette syndrome

We performed ALE meta-analysis to assess whether the identified coordinates (*n* = 77) of significant structural alterations in Tourette syndrome demonstrated spatial convergence. The analysis failed to identify any significant consistent findings at threshold (FWE *P* < 0.05). Exploratory analyses with coordinates split between higher and lower volume in Tourette syndrome patients identified a cluster of consistent significantly higher volume amongst patients involving the left thalamus and midbrain (centre of gravity *xyz* = −1.9, −12.7, −9.6; ALE value = 0.018; Supplementary Fig. 10). Only 2/6 studies^19,67^ contributed to this finding. (See Supplementary material and Supplementary Table 2 for further detail and exploratory analyses.)

Next, CNM was performed to assess whether this method would result in higher convergence of brain regions across studies. Despite coordinates being located in multiple different brain regions, all combined study seeds were functionally connected to a common brain network (Fig. 4D). Specifically, all seven combined study seeds were functionally connected (positively correlated) to the bilateral thalamus, midbrain and insular cortices. Six of the seven combined study seeds were connected (positively correlated) to the bilateral basal ganglia, cingulate gyrus and cerebellum. All combined study seeds were connected (negatively correlated) to small clusters within the right occipital fusiform gyrus and left superior lateral occipital cortex. Additionally, 6/7 combined study seeds were connected (negatively correlated) to the bilateral precuneus. (See Supplementary Figs. 11 and 12 for all identified voxels.)

### Mapping a network for tics in Tourette syndrome

A conjunction analysis was performed to show brain regions common to the ‘sensitive and specific’ lesion (Fig. 4C) and coordinate (Fig. 4D) networks and to define a tic network relevant to patients with Tourette syndrome. This network was characterized by positive connectivity to the bilateral posterior putamen, caudate nucleus and globus pallidus externus (GPe) and negative connectivity to the left precuneus (Fig. 4E).

### Network validation in Tourette syndrome patients

To validate this conjunction network, we then tested whether connectivity from this network is abnormal in patients with idiopathic Tourette syndrome. Positive and negative connectivity clusters derived from this conjunction network were run as separate regions of interest, because positively and negatively connected regions may differ biologically.

**Figure 5:**
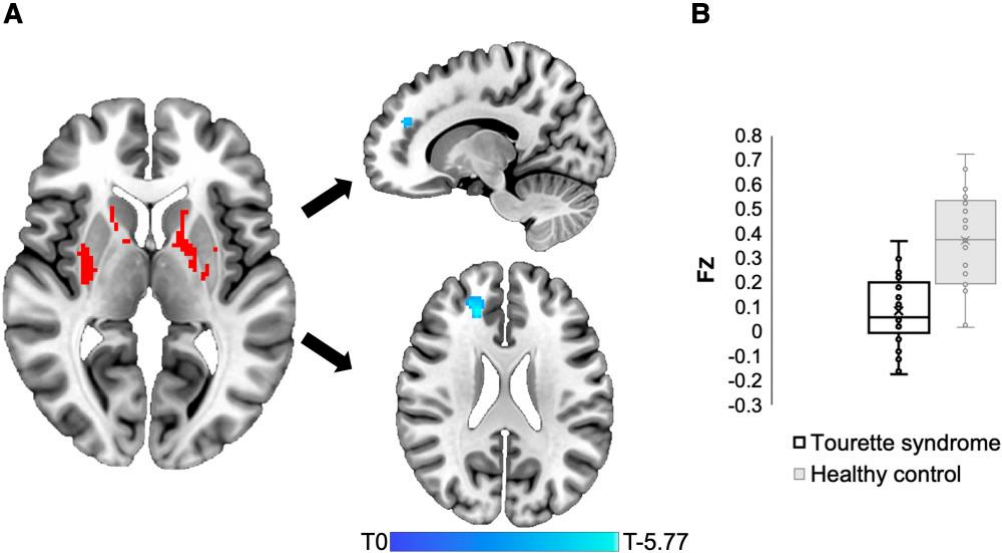
**Network validation in Tourette syndrome patients.** (**A**) The positive conjunction network for tics (left) derived from the LNM and CNM analyses. Using a GLM, this ‘seed’ demonstrated significantly lower positive connectivity in idiopathic Tourette syndrome patients (*n* = 21) compared to healthy controls (*n* = 25) to frontal and cingulate regions (right; *x* = 13.5, *z* = 23). Figures shown at FWE cluster-size corrected *P* < 0.05. Heat bar shows the *t-*value at each voxel when comparing patients and controls, demonstrating a peak value of −5.77 (*P* < 0.001). (**B**) The box and whisker plots represent the Fisher *z*-transformed (Fz) connectivity values for each group, with the x and middle line within the plot representing the mean and median, respectively.

The positive seed from the conjunction network for tics, involving the bilateral posterior putamen, caudate nucleus and GPe, demonstrated significantly abnormal connectivity to a cluster within the right frontal white matter extending into the cingulate gyrus, defined by lower positive connectivity in patients (peak MNI *xyz* = 18, 40, 26; cluster size = 99; cluster-size *P*_FWE_ = 0.037; Fig. 5A and B).

No significant differences in functional connectivity were identified between Tourette syndrome patients and controls from the negative conjunction seed, involving the left precuneus, nor from any seeds from the control networks from other movement disorders (*n* = 4, as there were positive and negative seeds for cervical dystonia). A post-hoc analysis demonstrated no significant differences in connectivity from our sensitive and specific LNM network (Fig. 4C). This demonstrates that the use of CNM was able to refine this lesion-derived network for tics and increase its relevance to patients with Tourette syndrome.

There was no significant difference in average in-scanner motion between patients and controls, based on framewise displacement (*P* = 0.078).

## Discussion

There are several important findings in this study. First, we independently identified a brain network for lesion-induced tics using LNM, predominantly involving the basal ganglia, largely consistent with the previous LNM study.^40^ Second, the combination of LNM with a similar mapping technique, using structural alterations from prior neuroimaging studies, effectively localized a network for tics relevant to patients with Tourette syndrome, encompassing the posterior putamen, caudate nucleus, GPe and precuneus. Finally, we demonstrated that connectivity from the positive network (involving the basal ganglia) was abnormal in a separate rs-fcMRI data set from patients with Tourette syndrome. The cortical cluster identified here in the precuneus provides a novel finding and may also offer an exciting opportunity for non-invasive brain stimulation (NIBS) protocols. Subcortical regions of this network closely resemble those identified by Ganos *et al*.,^40^ presenting as potential targets for invasive neuromodulation.

### Lesion and coordinate network mapping of neurological symptoms

To date, LNM has been used to localize brain networks in over 40 neurological disorders and symptoms.^38^ However, a limitation of this technique is that it is often difficult to quantify the extent to which these lesion-derived networks identify the same brain regions that are abnormal in their idiopathic counterparts. The CNM technique is useful in addressing this limitation as it uses coordinates of significant structural differences between idiopathic patients and healthy controls in a given disorder.^41–44^ This is of therapeutic relevance given that idiopathic populations comprise the majority of cases in most conditions, including tic disorders. As existing and future treatment solutions will predominantly be applied within the idiopathic population, combining these network mapping methods may provide greater utility in guiding therapeutic interventions. Here, we demonstrated the first combination of these two mapping techniques to refine a lesion network and improve its translation to an idiopathic disorder.

### Localization of lesion-induced tics

The lesions causing tics identified by our systematic search were mostly located within the basal ganglia (*n* = 13), yet were also distributed throughout multiple brain regions, such as the frontal lobes and cerebellum. When applying LNM, we found that the lesion locations were functionally connected to a common brain network. Despite performing LNM in tics independently from Ganos *et al*.^40^ and using more stringent criteria for the inclusion of lesions, the analyses localized consistent brain regions (Supplementary Fig. 1). Specifically, the present analysis identified a network with predominant functional connectivity to the basal ganglia, as well as the insular cortices, cingulate gyrus, thalamus, midbrain and cerebellum. Most of the lesions within our analysis (*n* = 11/19) were consistent with Ganos *et al*.,^40^ which in part accounts for the similar results. However, it is encouraging that the eight lesions not included by Ganos *et al*.^40^ also mapped to similar brain regions and demonstrates that their LNM findings are replicable. These regions have previously been implicated in the neural signature of tics, including the sensory phenomena preceding their occurrence.^23,24,26,68^ Functional neuroimaging investigations of tic expression have revealed an extensive network of brain regions activated prior to tic onset,^23,24^ including the insular and anterior cingulate cortices, putamen and thalamus, which may reflect the unpleasant urges suggested to drive tic generation.^68^ Supporting this proposal, functional abnormalities of the insular cortex have been associated with greater severity of premonitory urges.^26^ Previous functional and anatomical findings suggest that the cerebellum may also play a central role in the initiation and generation of tics.^23,69,70^ Excessive activity within the cerebellum, including a region identified in the present lesion network (lobule VI), was observed during the release of tics.^69^ Further, lower cerebellar volume has also been associated with greater tic severity.^70^

### Mapping a network for tics in Tourette syndrome

Acquired tics, such as those induced by a focal brain lesion, share similar clinical features to idiopathic tics seen in Tourette syndrome.^30^ However, the extent to which these symptoms activate congruent networks is largely unknown. Using structural alterations from prior neuroimaging studies to refine a lesion-induced tic network, we localized a network for tics relevant to patients with Tourette syndrome, encompassing the basal ganglia and precuneus.

Of note, we observed novel negative connectivity to the precuneus. While only a small number of voxels survived all LNM and CNM analyses, negative connectivity to this region was present within each analysis (Fig. 4). Although still a matter of debate, negative connectivity may reflect brain structures that are suppressed during the activation of competing regions.^71,72^ Negative connectivity to cortical regions has previously been shown in LNM of cervical dystonia^32^ and CNM of migraine.^42^ Corp *et al*.^32^ suggested that lesions could result in a loss of normal suppressive input from the lesion location to the region of negative connectivity. In the present context, a lesion causing tics could result in a loss of normal suppressive input from the lesion location to the precuneus and therefore hyperactivity in this region. The same interpretation could be made for coordinates of structural atrophy in patients,^42^ which may also result in loss of suppressive input from this tissue to the precuneus. A recent task-based fcMRI study examining the influence of emotion on the urge to tic in individuals with Tourette syndrome showed that greater activity within the precuneus was positively associated with premonitory urge severity.^73^ Further, using electroencephalography and network-based statistics, the left precuneus was identified as a hub within a network demonstrating increased connectivity during tic suppression.^74^ Given the role of the precuneus in self-awareness,^75^ this may reflect a neural substrate of premonitory urge.

Together, these findings may support the proposed hyperactivity of the precuneus in this population and its involvement in premonitory urge. However, Tourette syndrome patients did not show abnormal connectivity from the negative tic network involving the precuneus in the present study. As the precuneus has been implicated in premonitory urge,^73,74^ this non-significant finding may be due to the fact that we used rs-fcMRI to examine functional connectivity from this region. A task-based fcMRI paradigm measuring functional activity during tic suppression may be more sensitive to the possible abnormality of the precuneus in Tourette syndrome patients. It should also be noted that some of the coordinates included in our CNM analysis also represented higher grey and white matter volume in patients compared to controls. It is possible that structural abnormalities in general could result in a disruption of this normal negative connectivity and, thus, hyperactivity of this region. Alternatively, the effect on this region may be more complex than a simple increase in neural activity.

Our findings support evidence from neuroimaging and neuropathology studies favouring the basal ganglia as a key structure in tic pathophysiology.^76^ Involvement of the putamen in this network, specifically the posterior portion, is interesting given this structure’s prominent role in movement control. This finding may be consistent with evidence of excessive dopamine release within the putamen in individuals with Tourette syndrome.^77^ Indeed, structural abnormalities of this region have been suggested to reflect the dopaminergic dysfunction implicated in Tourette syndrome.^78^ Further, seminal and prospective studies in patients with Tourette syndrome implicate abnormalities of the caudate nucleus as a morphological trait marker of tics,^79,80^ with lower volume of the caudate nucleus in childhood being predictive of greater tic severity in early adulthood.^80^ Hyperactivity of the GPe and prefrontal regions underlying Tourette syndrome has been proposed.^81^ Promising findings in several patients receiving deep brain stimulation (DBS) to the GPe also support this structure’s role in tic generation.^81,82^

One of the most noteworthy findings is the demonstration that connectivity from the positive network for tics, derived from the conjunction between lesion-induced and idiopathic data, was abnormal in a separate rs-fcMRI data set from patients with idiopathic Tourette syndrome. Tics are proposed, in part, to emerge from the dysfunction of circuits that link the striatum and frontal cortex.^9,10^ Here, we found that children and adolescents with Tourette syndrome show lower functional connectivity compared to healthy controls between this network (involving the basal ganglia) and a cluster within the frontal white matter extending into the cingulate gyrus. A previous rs-fcMRI study using graph theoretical analysis showed that functional connectivity within the cortico-striato-thalamo-cortical (CSTC) circuits was disorganized in adults with Tourette syndrome. Specifically, abnormal connectivity between the cortex and basal ganglia was reported,^83^ which may be consistent with the present findings. Moreover, a previous study examining volume and microstructure in Tourette syndrome found that lower white matter of the right frontal pole was the only significant volumetric correlate of tic severity in patients.^15^ Given that this network was selectively abnormal compared to control regions of interest from other movement disorders, this supports the specificity of this network to Tourette syndrome. Interestingly, while connectivity from the positive conjunction network for tics was abnormal in patients with idiopathic Tourette syndrome, connectivity did not significantly differ between patients and controls from regions sensitive and specific to the present lesion-induced tic network. This finding demonstrates that the use of CNM was able to refine this lesion-derived network for tics and increase its relevance to patients with Tourette syndrome.

### Potential targets for neuromodulation in Tourette syndrome

Despite existing support for the efficacy of DBS for refractory tics, there are ethical considerations that limit its utility in this clinical population.^84^ As a neurodevelopmental disorder, peak severity of Tourette syndrome symptoms is typically experienced during early adolescence, around 10–12 years of age.^85,86^ However, the application of DBS for severe medically intractable tics in the paediatric population remains controversial.^87^ To date, a small number of cases support the efficacy of DBS in children with refractory tics;^88^ however, there is currently limited data on the long-term clinical outcomes of this treatment in these patients.^84^ Over the past two decades, the utility of NIBS as a treatment for tics has been assessed and shown to be safe and effective in children with Tourette syndrome.^89–91^ Previous functional neuroimaging studies have demonstrated that regions of the parietal lobe are active prior to tic onset,^23,24^ making this structure a candidate region for NIBS in this population. Recently, a randomized double-blind sham-controlled trial in patients 15–30 years old targeted the parietal lobe (P3 and P4 electrode sites) with low-frequency repetitive transcranial magnetic stimulation (rTMS). Significant reductions in motor and vocal tic severity and premonitory urge were reported, which were maintained for at least 1 month.^92^ Although the precuneus is yet to be trialled in Tourette syndrome to our knowledge, a double-blind randomized placebo-controlled trial applied rTMS to this region in patients with Alzheimer’s disease, ameliorating cognitive decline.^93^ Further, computational modelling has shown a substantial induced electric field within the precuneus using transcranial alternating current stimulation in patients with Alzheimer’s disease.^94^ In Tourette syndrome, low-frequency rTMS could have an inhibitory effect on this region, which may improve tic symptoms. Together, these findings support this structure’s role in the modulation of tic severity and provide a testable target for future NIBS protocols.

Pharmacological therapy is the current first-line treatment for moderate-to-severe tics.^95^ However, recent estimates suggest that ∼30% of adults with Tourette syndrome demonstrate moderate-to-severe tics that are unresponsive to non-invasive treatments, with such cases being eligible for DBS.^96^ Since the initial application of DBS in Tourette syndrome in 1999,^97^ over 200 patients have undergone this invasive treatment, reporting an average 40% improvement in tic symptoms.^98–100^ While the optimal DBS target for tics is debated, based on evidence of CSTC circuit dysfunction, regions of the GPi and thalamus (e.g. centromedian nucleus) are the most common targets for invasive neuromodulation in patients with Tourette syndrome.^82,100^ Using computational models of the volume of tissue activated in a retrospective sample of patients that underwent DBS for treatment-refractory tics, Johnson *et al*.^101^ found that stimulation of the GPi extended laterally into the GPe, further supporting the relevance of this structure in tic generation. The thalamic regions of the present LNM and CNM networks closely resemble those identified in the previous LNM study.^40^ Ganos *et al*.^40^ showed that connectivity between DBS electrodes within the thalamus and their network for lesion-induced tics was predictive of tic symptom improvement. These findings may inform optimal connectivity profiles for neuromodulation to improve tic symptoms.

### Limitations

There are some limitations that should be acknowledged. First, a relatively small sample of studies met our predefined inclusion criteria for the ALE and CNM analyses (*n* = 7). It is possible that this may have contributed to the limited convergence established using standard ALE meta-analytic methods.^43^ Similarly, we applied one meta-analytic technique (ALE) to our data, while others have been applied to Tourette syndrome and could reveal different findings.^29^ However, using CNM, we identified 100% convergence in multiple cortical and subcortical structures that have previously been implicated in Tourette syndrome. Second, there are potential limitations of the LNM method, including using a normative connectome to map neuropsychiatric symptoms and using manually traced 2D rather than 3D lesions as inputs. Nonetheless, this technique has been applied in multiple clinical populations with findings predictive of clinical improvement.^32–34,40^ Third, we relied on the clinical judgment of the original authors of the case reports, including their descriptions, diagnoses and examinations of tic aetiology, rather than direct observation. It is possible that some relevant aspects of symptom presentation were not reported, which may introduce noise into the analysis due to clinical heterogeneity. Fourth, we acknowledge that while normalization to standard space is important for pre-processing and analyzing fcMRI data, performance can vary in the paediatric population. Fifth, the publicly available data set used to validate the identified network in patients with idiopathic Tourette syndrome did not collect tic or premonitory urge severity scores as part of its protocol, given that it was not specific to tic disorders. Future validation of this network in a separate functional data set with access to behavioural data would be useful in examining the relationship between network connectivity and patient symptoms. Finally, this data set was acquired from children and adolescents. It is possible that age-related differences in Tourette syndrome functional networks^102^ may account for the non-significant difference in connectivity from the negative network for tics derived from lesions and coordinates, which comprised both children and adults.

## Conclusions

Consistent with the previous LNM study,^40^ lesions causing tics localized to a common neural network, predominantly involving the basal ganglia. Using structural brain alterations from prior neuroimaging studies, we refined this lesion-induced tic network and localized a network for tics relevant to patients with Tourette syndrome. This network is defined by connectivity to the posterior putamen, caudate nucleus, GPe and precuneus. Functional connectivity from the positive network (involving the basal ganglia) to frontal and cingulate regions was abnormal in patients with idiopathic Tourette syndrome, validating its relevance in this population. Finally, we reported a novel finding of negative cortical connectivity to a cluster within the precuneus, providing a testable target for NIBS protocols.

## Supplementary Material

fcad105_Supplementary_Data

## Data Availability

Patient phenotypic data used in the rs-fcMRI analyses is available through data agreement with the HBN (http://fcon_1000.projects.nitrc.org/indi/cmi_healthy_brain_network/Pheno_Access.html). Raw de-identified neuroimaging data is available from the HBN database (http://fcon_1000.projects.nitrc.org/indi/cmi_healthy_brain_network/sharing_neuro.html). The unthresholded LNM and CNM maps and binary conjunction networks are available from NeuroVault (https://neurovault.org/collections/PAQBHGRK/).^66^ Lesion tracings, lesion and combined study seed *t*-maps and the LNM and CNM analysis code are available upon request from the corresponding authors.

## Abbreviations

ALE =: anatomical likelihood estimation
BOLD =: blood-oxygen-level-dependent
CNM =: coordinate network mapping
CONN =: functional connectivity toolbox
CSTC =: cortico-striato-thalamo-cortical
DBS =: deep brain stimulation
FSL =: Functional Magnetic Resonance Imaging of the Brain Software Library
FWE =: family-wise error
GLM =: general linear model
GPe =: globus pallidus externus
GPi =: globus pallidus internus
HBN =: Healthy Brain Network
ICA-AROMA =: independent component analysis automatic removal of motion artefacts
LNM =: lesion network mapping
MNI =: Montreal Neurological Institute
NIBS =: non-invasive brain stimulation
rs-fcMRI =: resting-state functional connectivity MRI
rTMS =: repetitive transcranial magnetic stimulation
