# Supplementary material for: Mapping a network for tics in Tourette syndrome using causal lesions and structural alterations
Source: Brain Commun. 2023 Apr 4;5(3):fcad105. doi: 10.1093/braincomms/fcad105 (PMC10198704; doi:10.1093/braincomms/fcad105)
Supplement: fcad105_Supplementary_Data [file fcad105_supplementary_data.zip › Supplementary_figures_and_legends.pdf]

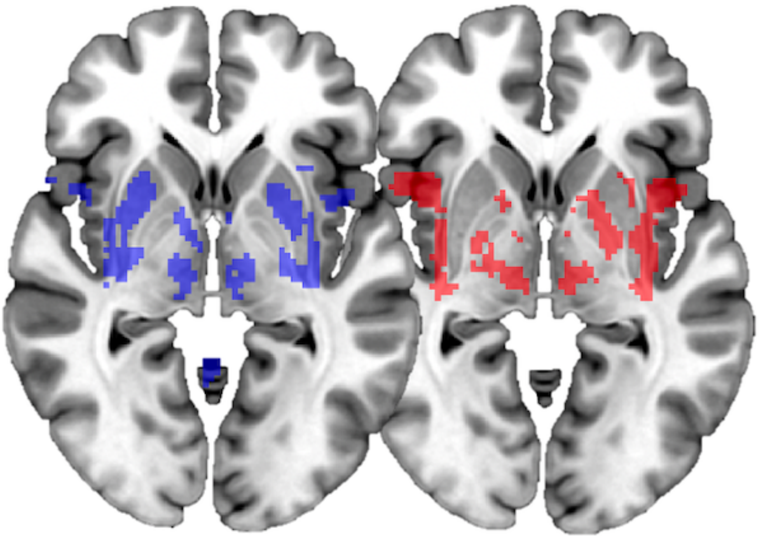

Supplementary Figure 1. Spatial convergence between lesion-induced tic networks. Spatial convergence between the positive network map for the present LNM analysis of lesion-induced tics (blue) and that of Ganos *et al.*[1] (red). These maps showed strong spatial convergence, with network overlap in the insular cortices, cingulate gyrus, basal ganglia, thalamus, and cerebellum (z = -2).

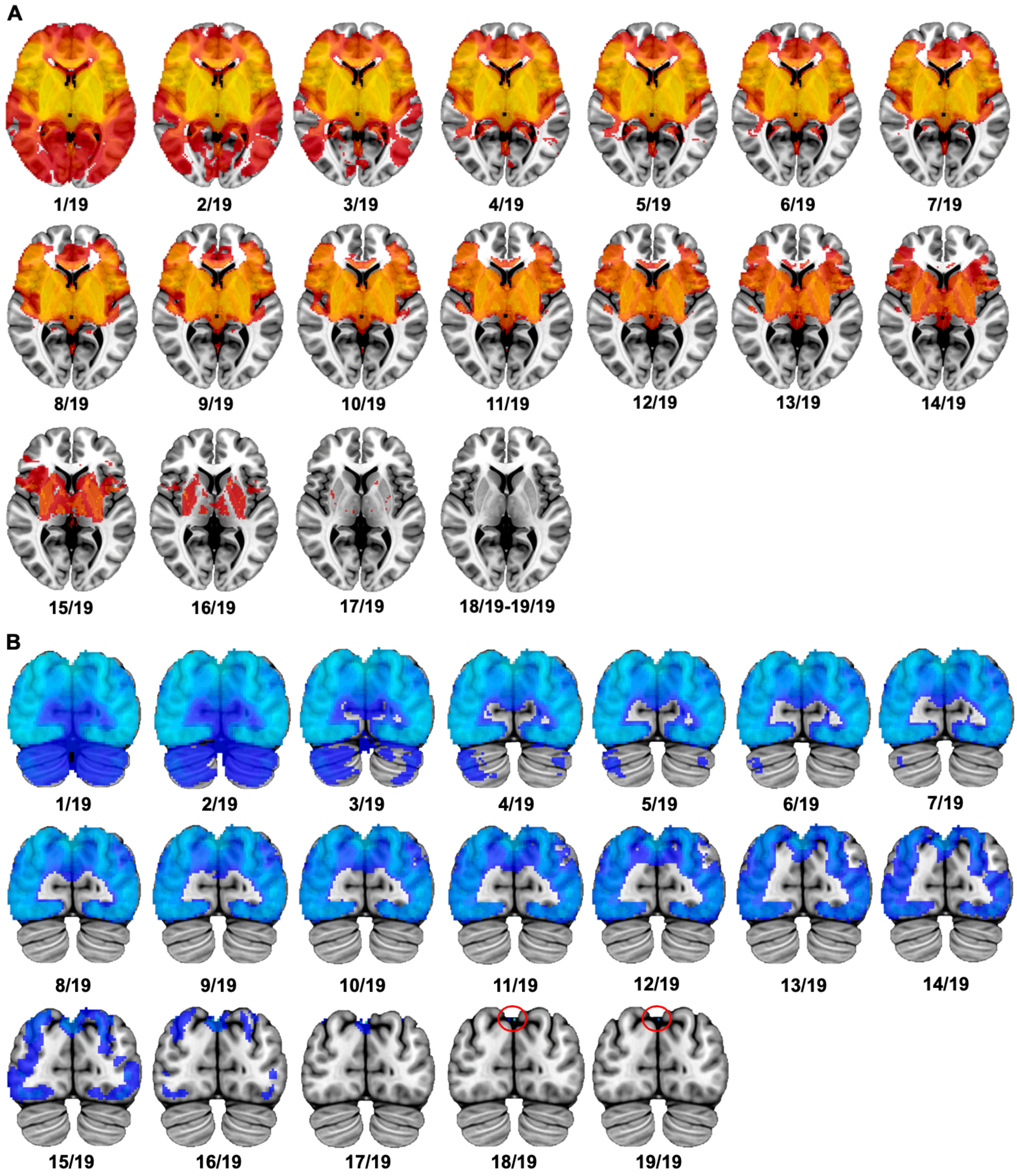

Supplementary Figure 2. (**A**) Positive lesion network map showing different distributions of voxels surviving various thresholds (z = 4). (**B**) Negative lesion network map showing different distributions of voxels surviving various thresholds (y = -81.5). Optimal and chosen threshold was identified as ≥ 16/19 lesions.

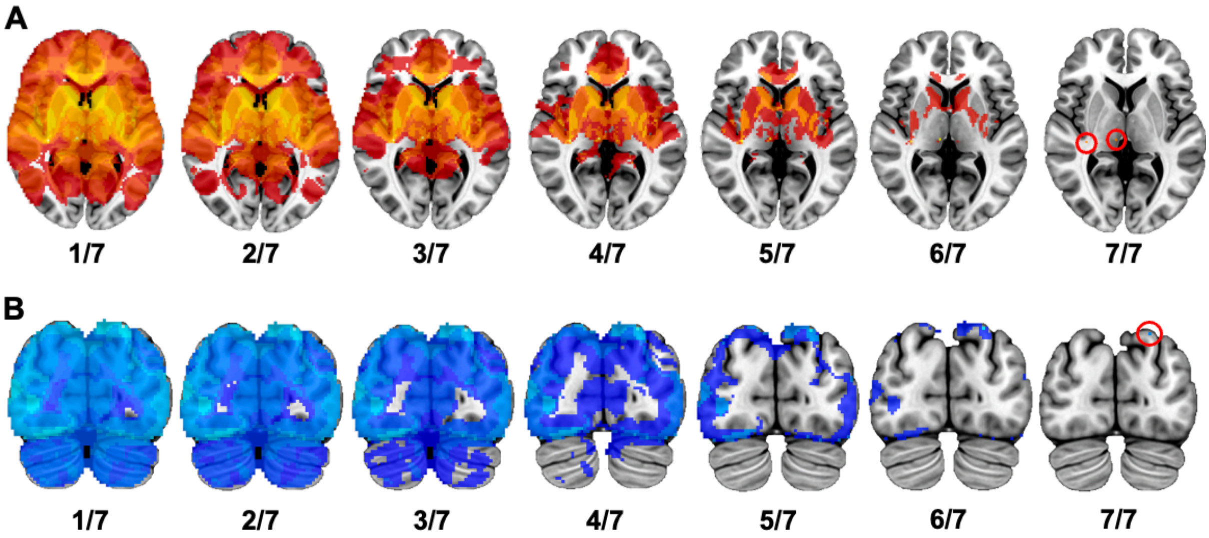

Supplementary Figure 3. (**A**) Positive coordinate network map showing different distributions of voxels surviving various thresholds (z = 4). (**B**) Negative coordinate network map showing different distributions of voxels surviving various thresholds (y = -81.5). Optimal and chosen threshold was identified as ≥ 6/7 combined study seeds.

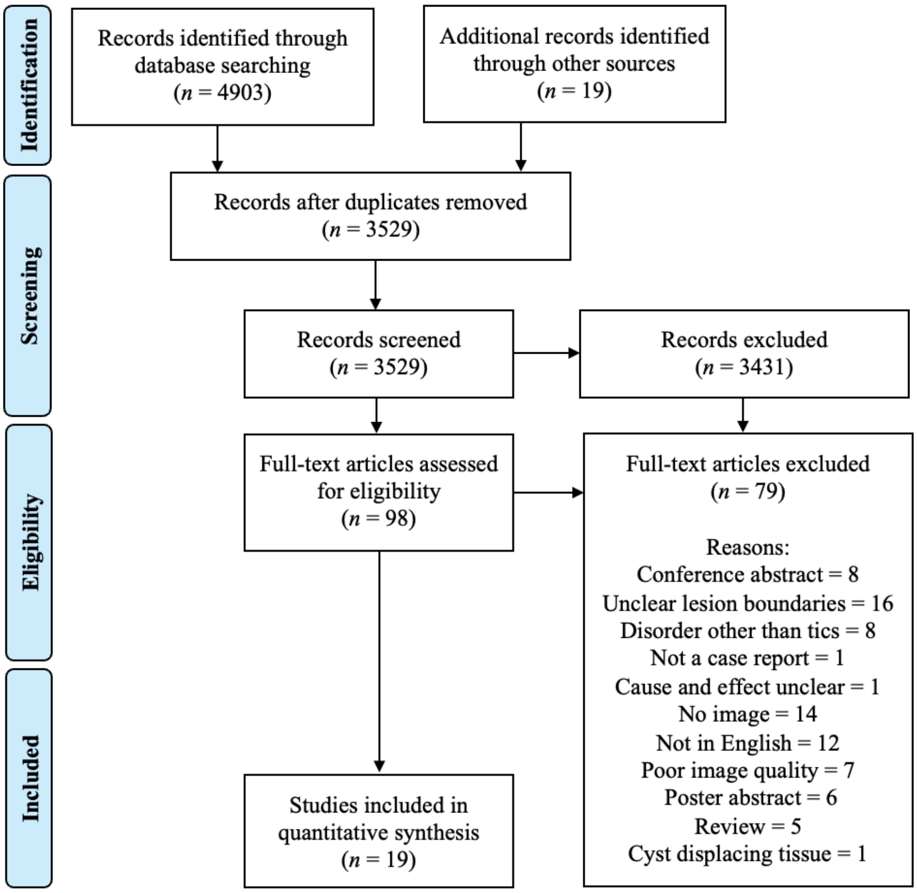

Supplementary Figure 4. Flowchart for the identification of included cases in the LNM analysis. For the LNM analysis, the initial search of PubMed and Embase yielded a total of 4,903 results, with 19 reports sourced from the reference lists of included cases and a previously published LNM analysis of lesion-induced tics[1] (n = 2). No limiters for publication status or year were applied, however, only reports in English were considered. As tics can appear many months following a brain injury,[2,3] a stringent time-limit for the occurrence of tics post-lesion was not applied. After the removal of duplicates, titles and abstracts of 3,529 articles were screened. Following full-text screening against the inclusion criteria, 19 cases were included in the final analysis.

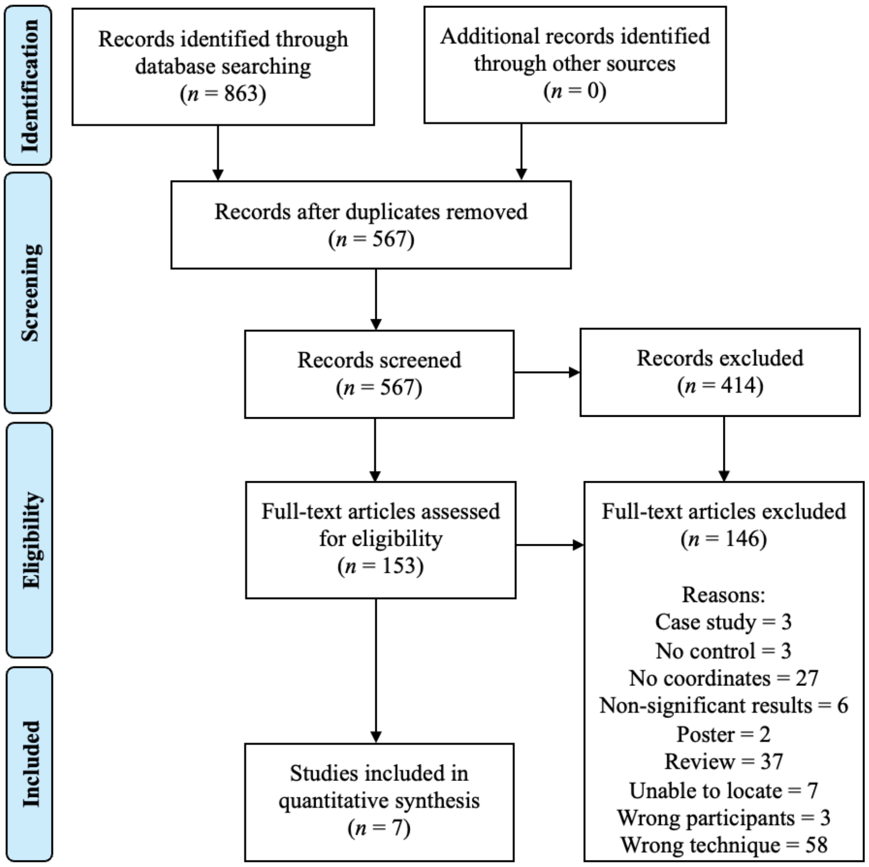

Supplementary Figure 5. Flowchart for the identification of included studies in the ALE and CNM analyses. For the ALE and CNM analyses, the initial search of MEDLINE Complete and Embase yielded 863 studies. No limiters for publication status or year were applied, however, only studies in English were considered. Studies examining neurotransmitter activity were excluded to prevent biased findings in brain regions that are dense in specific neurotransmitters. After the removal of duplicates, and screening of titles and abstracts, 153 full-text articles were assessed for eligibility, with seven studies included in the final analyses. The systematic search was updated in August 2021 (limiting to studies published between 2019-2021), however, no additional articles were included in the analyses.

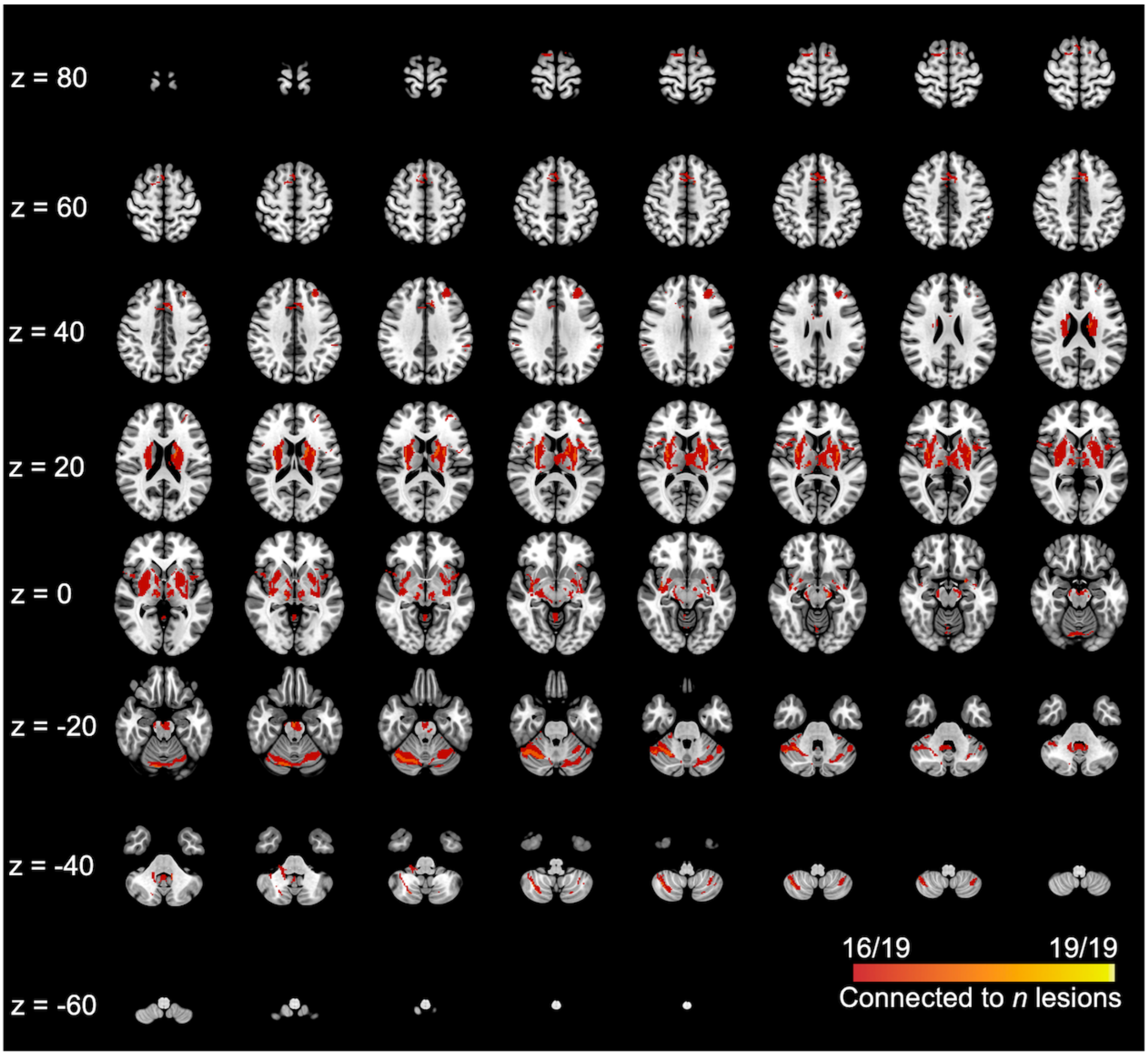

Supplementary Figure 6. Lesion-induced tic network at optimal threshold – positive map. Axial slices showing brain regions positively correlated to lesions causing tics, thresholded at ≥ 16/19 lesions.

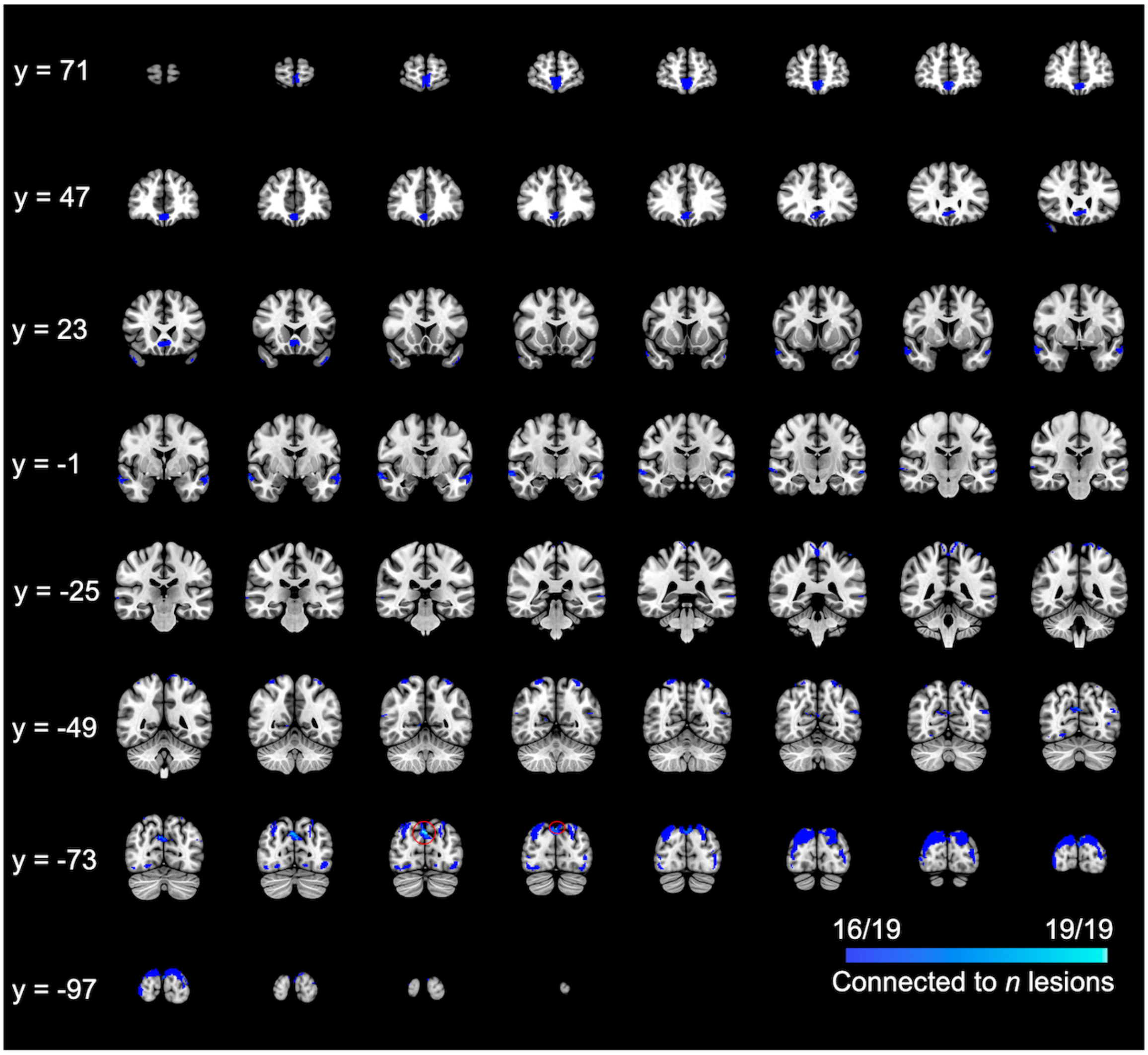

Supplementary Figure 7. Lesion-induced tic network at optimal threshold – negative map. Coronal slices showing brain regions negatively correlated to lesions causing tics, thresholded at ≥ 16/19 lesions.

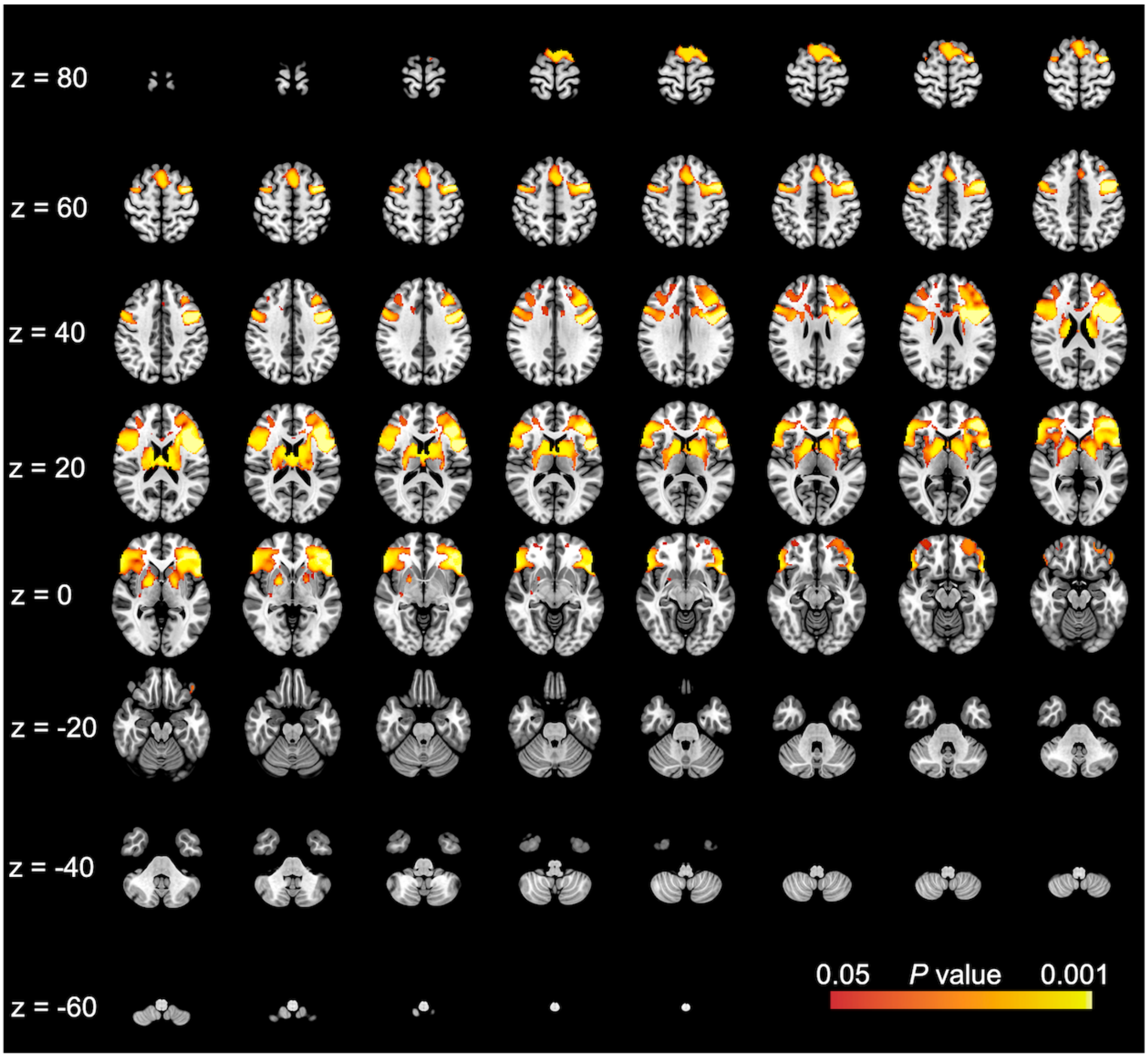

Supplementary Figure 8. Brain regions 'specific' to lesion-induced tics – positive map. Axial slices showing voxels specific to lesions causing tics compared to those causing other movement disorders (cervical dystonia, parkinsonism, Holmes tremor).

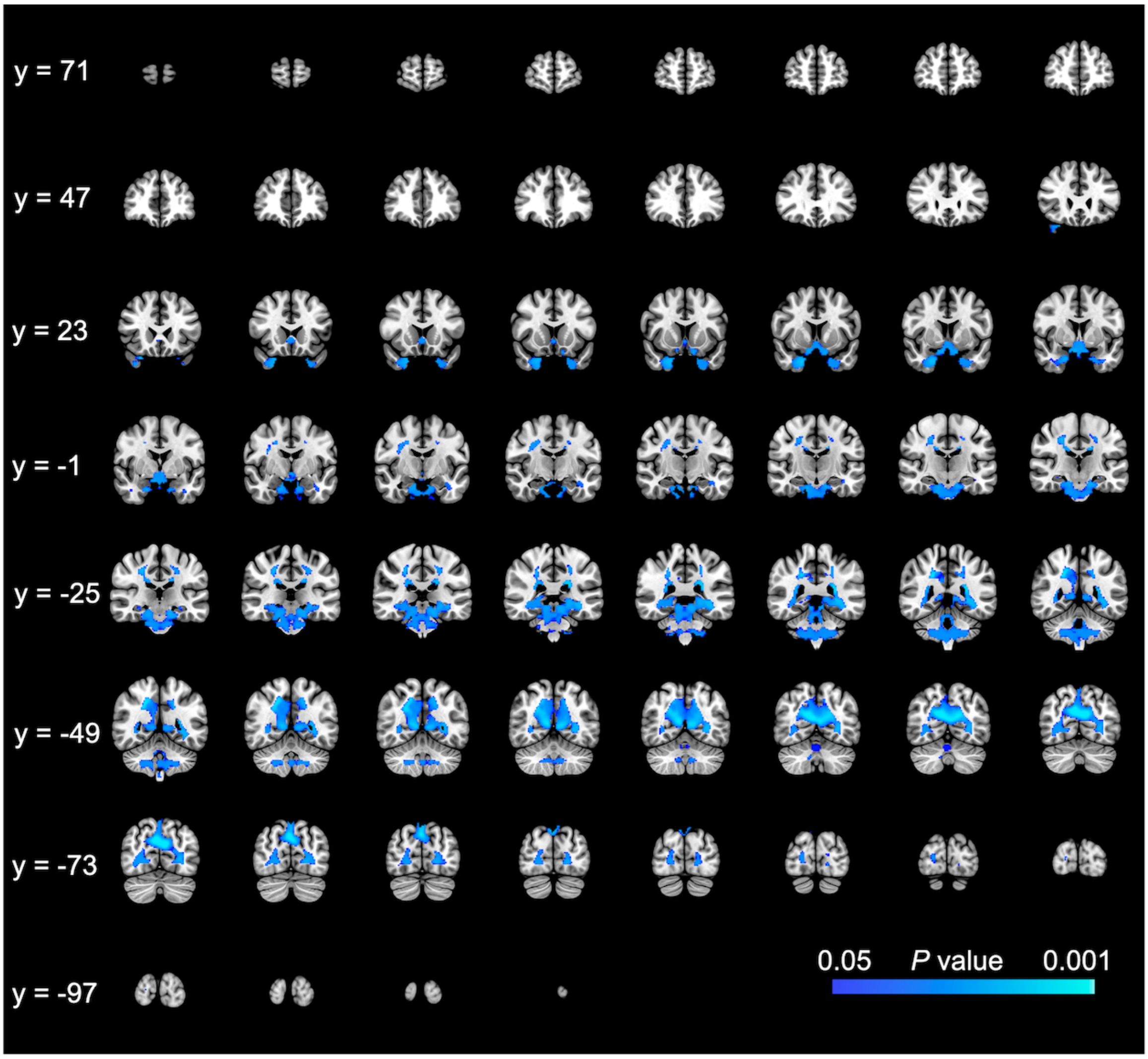

Supplementary Figure 9. Brain regions 'specific' to lesion-induced tics – negative map. Coronal slices showing voxels specific to lesions causing tics compared to those causing other movement disorders (cervical dystonia, parkinsonism, Holmes tremor).

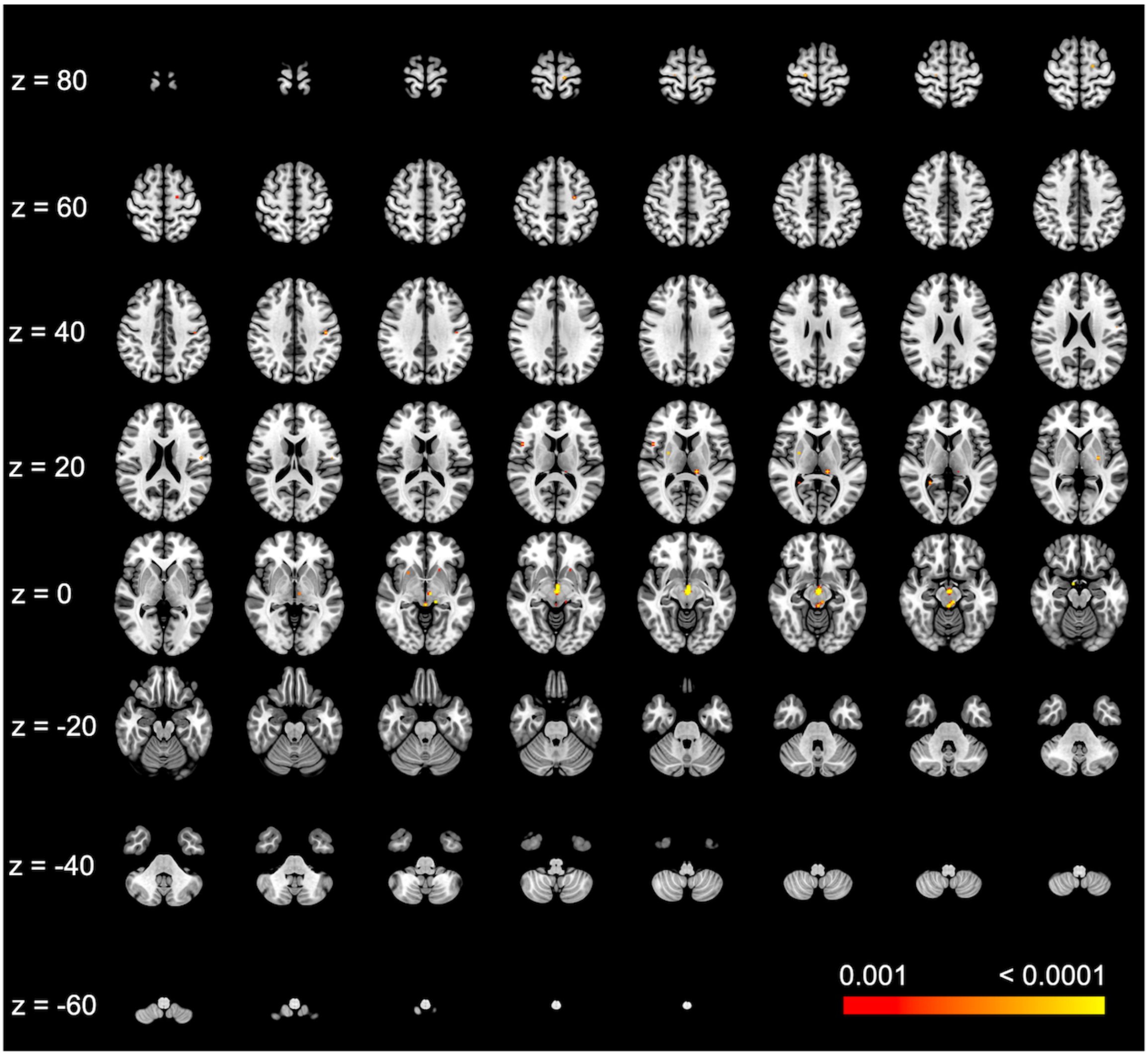

Supplementary Figure 10. Consistent regions of abnormality in Tourette syndrome. Exploratory anatomical likelihood estimation meta-analysis of higher volume in Tourette syndrome (presented using uncorrected *P* threshold < 0.001 for visualization purposes[4]). Only 2/6 studies[5,6] contributed to the significant consistent finding at threshold.

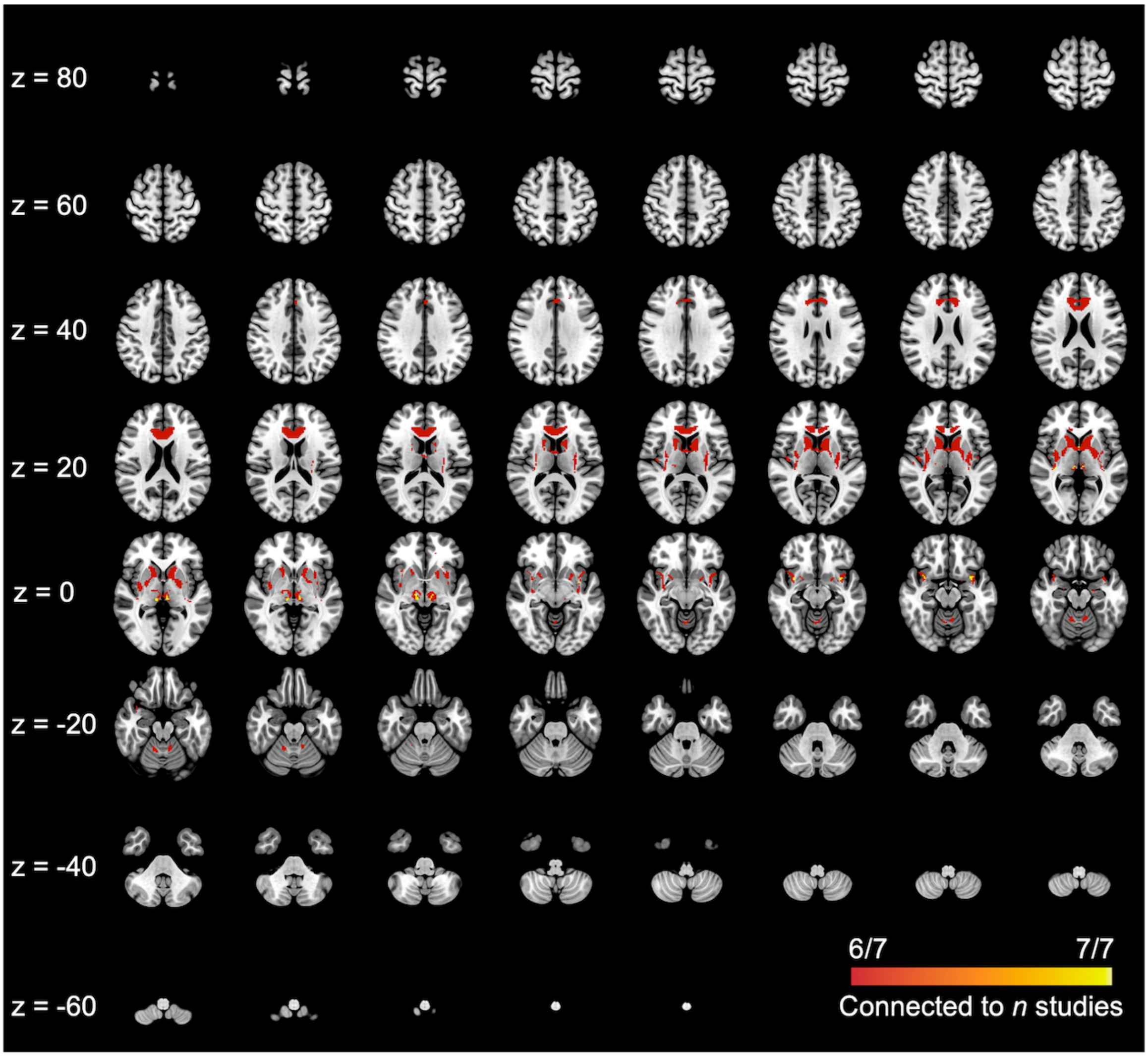

Supplementary Figure 11. Coordinate network for Tourette syndrome at optimal threshold – positive map. Axial slices showing brain regions positively correlated to the combined seeds from the included studies, thresholded at ≥ 6/7 combined study seeds.

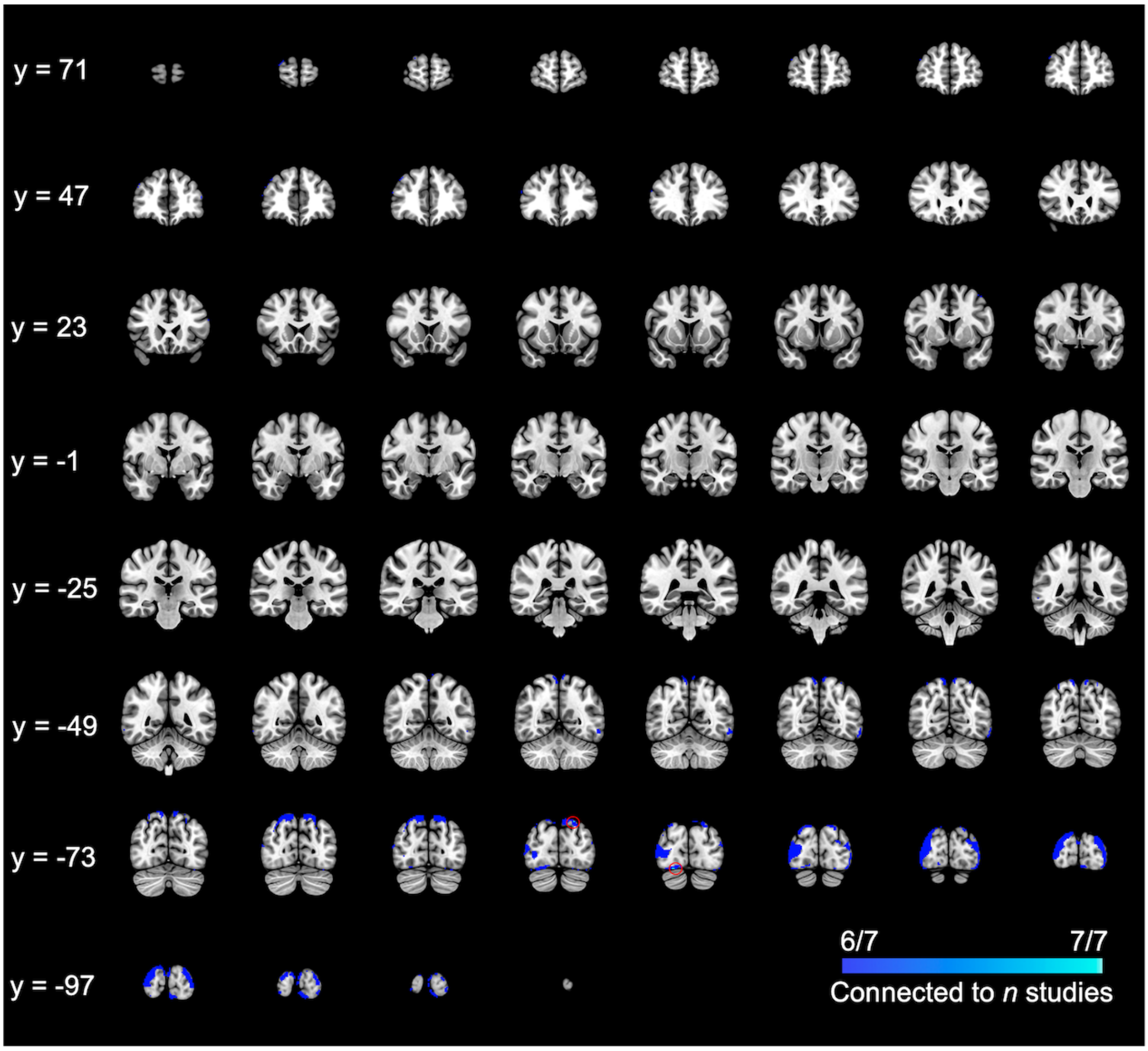

Supplementary Figure 12. Coordinate network for Tourette syndrome at optimal threshold – negative map. Coronal slices showing brain regions negatively correlated to the combined seeds from the included studies, thresholded at ≥ 6/7 combined study seeds.

References

1. Ganos C, Al-Fatly B, Fischer JF, et al. A neural network for tics: insights from causal brain lesions and deep brain stimulation. *Brain*. Published online January 13, 2022:awac009. doi:10.1093/brain/awac009
2. Krauss JK, Jankovic J. Tics secondary to craniocerebral trauma. *Mov Disord*. 1997;12(5):776-782. doi:10.1002/mds.870120527
3. Ranjan N, Nair KPS, Romanoski C, Singh R, Venketswara G. Tics after traumatic brain injury. *Brain Inj*. 2011;25(6):629-633. doi:10.3109/02699052.2011.572944
4. Fuelscher I, Caeyenberghs K, Enticott PG, Williams J, Lum J, Hyde C. Differential activation of brain areas in children with developmental coordination disorder during tasks of manual dexterity: An ALE meta-analysis. *Neurosci Biobehav Rev*. 2018;86:77-84. doi:10.1016/j.neubiorev.2018.01.002
5. Greene DJ, Williams III AC, Koller JM, Schlaggar BL, Black KJ, and The Tourette Association of America Neuroimaging Consortium. Brain structure in pediatric Tourette syndrome. *Mol Psychiatry*. 2017;22(7):972-980. doi:10.1038/mp.2016.194
6. Garraux G, Goldfine A, Bohlhalter S, Lerner A, Hanakawa T, Hallett M. Increased midbrain gray matter in Tourette's syndrome. *Ann Neurol*. 2006;59(2):381-385. doi:10.1002/ana.20765
