# Supplementary material for: Mapping a network for tics in Tourette syndrome using causal lesions and structural alterations
Source: Brain Commun. 2023 Apr 4;5(3):fcad105. doi: 10.1093/braincomms/fcad105 (PMC10198704; doi:10.1093/braincomms/fcad105)
Supplement: fcad105_Supplementary_Data [file fcad105_supplementary_data.zip › Supplementary_material.pdf]

Supplementary material

Search syntax used for the lesion network mapping (LNM) analysis

Embase

S1: lesion*:ab,ti,kw OR stroke*:ab,ti,kw OR infarct*:ab,ti,kw OR ischem*:ab,ti,kw OR ischaem*:ab,ti,kw OR hemorrhag*:ab,ti,kw OR haemorrhag*:ab,ti,kw OR tumor*:ab,ti,kw OR tumour*:ab,ti,kw OR plaque*:ab,ti,kw OR 'brain injur*':ab,ti,kw

S2: tourett*:ab,ti,kw OR tic:ab,ti,kw OR tics:ab,ti,kw OR 'gilles de la tourette syndrome'/de

S3: S1 AND S2

Limitations: search Embase only

PubMed

S1: lesion* OR stroke* OR infarct* OR ischem* OR ischaem* OR hemorrhag* OR haemorrhag* OR tumor* OR tumour* OR plaque* OR "brain injur*"

S2: Tourette Syndrome[MeSH Terms] OR Tic Disorders[MeSH Terms] OR tourett* OR tic OR tics

S3: S1 AND S2

Spatial correlation between lesion-induced tic networks

To measure the spatial correlation between the present and previously published LNM, we performed several analyses following validated methods (see Siddiqi *et al.*[1]). After finalizing our LNM analysis, we received the functional connectivity *t*-maps of lesions used by Ganos *et al.*[2]. First, we computed spatial correlations between the *t*-maps of lesions that were consistent (n = 11) across both datasets. This was done by correlating *t*-maps for the same lesion across both datasets and then averaging the correlations for all lesion pairs. Next, we assessed the spatial correlation between the *t*-maps that were unique to our dataset (n = 8) and that of Ganos *et al.*[2] (n = 11). Strong spatial correlation was shown between shared lesions (n = 11, r = 0.868) confirming similar lesion tracings. Comparison of lesions that were unique to our dataset (n = 8) and that of Ganos *et al.*[2] (n = 11) produced a spatial correlation of r = 0.344. Spatial convergence between the present and previously published LNM analyses is shown in Supplementary Fig. 1.

Search syntax used for the anatomical likelihood estimation (ALE) and coordinate network mapping (CNM) analyses

Embase

S1: MRI:ti OR "magnetic resonance imaging”:ti OR MRI:ab OR "magnetic resonance imaging”:ab OR VBM:ti OR VBM:ab OR "voxel based morpho*”:ti OR "voxel based morpho*”:ab OR SPECT:ti OR SPECT:ab OR "single photon emission computed tomography”:ti OR "single photon emission computed tomography”:ab OR "positron emission tomography”:ti OR "positron emission tomography”:ab OR PET:ti OR PET:ab OR hypometabolism:ti OR hypometabolism:ab OR hypoperfusion:ti OR hypoperfusion:ab OR atrophy near/9 brain OR atrophy near/9 matter OR atrophy near/9 cort* OR atrophy near/9 neuro*

S2: 'gilles de la tourette syndrome'/de OR 'tourette* syndrome':ti OR 'tourette* syndrome':ab OR tourette*:ti OR tourette*:ab

S3: S1 AND S2

Limitations: search Embase only

MEDLINE Complete

S1: TI MRI OR TI "magnetic resonance imaging" OR AB MRI OR AB "magnetic resonance imaging" OR MH Magnetic Resonance Imaging OR TI VBM OR AB VBM OR TI "voxel based morpho*" OR AB "voxel based morpho*" OR TI SPECT OR AB SPECT OR TI "single photon emission computed tomography" OR AB "single photon emission computed tomography" OR MH Tomography, Emission-Computed, Single-Photon OR TI "positron emission tomography" OR AB "positron emission tomography" OR TI PET OR AB PET OR MH Positron-Emission Tomography OR TI hypometabolism OR AB hypometabolism OR TI hypoperfusion OR AB hypoperfusion OR (TI (atrophy) N9 (brain OR matter OR cort* OR neuro*)) OR (AB (atrophy) N9 (brain OR matter OR cort* OR neuro*))

S2: (MH "Tourette Syndrome") OR TI tourette* OR AB tourette* OR TI "tourette* syndrome" OR AB "tourette* syndrome”

S3: S1 AND S2

ALE meta-analysis in Tourette syndrome

We conducted ALE meta-analysis to test for regions of consistent significant structural differences, that is, higher or lower regional grey and white matter volume in Tourette syndrome patients relative to controls. Exploratory analyses were conducted using two contrasts. The first contrast examined regions showing higher volume in patients relative to controls, with the second testing for areas of lower volume amongst patients compared to controls. Analyses were performed following the current methods of Eickhoff *et al.*[3]. We used the revised algorithm,[3] which applies a random-effects model,[4] with refined permutation testing and stringent correction for multiple comparisons. The analyses were conducted with a cluster-forming threshold of $P < 0.001$ (uncorrected), 1000 permutations, and corrected for multiple comparisons using a cluster-level interference threshold of FWE $P < 0.05$.[3]

Seven studies reporting brain alterations in Tourette syndrome were included in the present ALE meta-analysis, all of which used voxel-based morphometry to measure regional grey and white matter volume across the whole brain. The analysis failed to identify any significant consistent findings at threshold (FWE $P < 0.05$). Exploratory analyses were conducted in two contrasts. The first contrast tested for consistently higher volume in Tourette syndrome and involved six studies (22 coordinates), excluding one article reporting only lower volume in patients. Only 2/6 studies[5,6] contributed to the significant consistent finding at threshold, involving the left thalamus and midbrain (center of gravity xyz = -1.9, -12.7, -9.6; ALE value = 0.018; Supplementary Fig. 10). The second contrast examined areas of consistently lower volume in patients and involved six studies (55 coordinates), excluding one article reporting only higher volume amongst patients. No significant regions of convergence were identified.

To replicate previous ALE meta-analytic findings (see Wen *et al.*[7]) in our included coordinates of brain alterations in Tourette syndrome, we conducted two further ALE meta-analyses in addition to those reported above. There was not exact overlap between the studies included in the present ALE meta-analyses and those of Wen *et al.*[7], as two studies involved in their analyses did not meet our inclusion criteria (i.e., did not use whole-brain analysis). Analyses were performed in two contrasts. The first contrast examined regions showing higher volume in patients relative to controls, with the second testing for areas of lower volume amongst patients compared to controls. As per the methods of Wen *et al.*[7], ALE maps were corrected for multiple comparisons using the false discovery rate ($P < 0.05$), with a cluster extent of 50mm^3. Using this method, no significant clusters were identified in our included coordinates

of brain abnormalities when volume was higher or lower in Tourette syndrome relative to controls.

As our replication ALE meta-analyses did not identify significant clusters in both higher and lower volume in Tourette syndrome compared to controls, we conducted two further exploratory analyses in an attempt to replicate the findings of Wen *et al.*[7] as closely as possible using our coordinates of brain alterations. ALE maps were thresholded at $P < 0.001$ (uncorrected), with a cluster extent of $100mm^3$. The results of these exploratory analyses are presented in Supplementary Table 2.

Neuroanatomical location of the clusters identified by the ALE meta-analyses was confirmed using the Harvard-Oxford cortical and subcortical structural atlases (FSL[8]; version 6.0.4).

References

1. Siddiqi SH, Schaper FLWVJ, Horn A, et al. Brain stimulation and brain lesions converge on common causal circuits in neuropsychiatric disease. *Nat Hum Behav*. 2021;5(12):1707-1716. doi:10.1038/s41562-021-01161-1
2. Ganos C, Al-Fatly B, Fischer JF, et al. A neural network for tics: insights from causal brain lesions and deep brain stimulation. *Brain*. Published online January 13, 2022:awac009. doi:10.1093/brain/awac009
3. Eickhoff SB, Bzdok D, Laird AR, Kurth F, Fox PT. Activation likelihood estimation meta-analysis revisited. *NeuroImage*. 2012;59(3):2349-2361. doi:10.1016/j.neuroimage.2011.09.017
4. Eickhoff SB, Laird AR, Grefkes C, Wang LE, Zilles K, Fox PT. Coordinate-based activation likelihood estimation meta-analysis of neuroimaging data: A random-effects approach based on empirical estimates of spatial uncertainty. *Hum Brain Mapp*. 2009;30(9):2907-2926. doi:10.1002/hbm.20718
5. Greene DJ, Williams III AC, Koller JM, Schlaggar BL, Black KJ, and The Tourette Association of America Neuroimaging Consortium. Brain structure in pediatric Tourette syndrome. *Mol Psychiatry*. 2017;22(7):972-980. doi:10.1038/mp.2016.194
6. Garraux G, Goldfine A, Bohlhalter S, Lerner A, Hanakawa T, Hallett M. Increased midbrain gray matter in Tourette's syndrome. *Ann Neurol*. 2006;59(2):381-385. doi:10.1002/ana.20765
7. Wen F, Yan J, Yu L, et al. Grey matter abnormalities in Tourette syndrome: an activation likelihood estimation meta-analysis. *BMC Psychiatry*. 2021;21(1):184. doi:10.1186/s12888-021-03187-1
8. Jenkinson M, Beckmann CF, Behrens TEJ, Woolrich MW, Smith SM. FSL. *NeuroImage*. 2012;62(2):782-790. doi:10.1016/j.neuroimage.2011.09.015
