# Supplementary material for: Mapping a network for tics in Tourette syndrome using causal lesions and structural alterations
Source: Brain Commun. 2023 Apr 4;5(3):fcad105. doi: 10.1093/braincomms/fcad105 (PMC10198704; doi:10.1093/braincomms/fcad105)
Supplement: fcad105_Supplementary_Data [file fcad105_supplementary_data.zip › Supplementary_tables_1-7.pdf]

Supplementary Table 1 Reasons for excluding cases of lesion-induced tics

Case	Reason for Exclusion
Dale[1]	Poor image quality. Clear lesion boundaries could not be discerned to accurately trace the lesion location.
Jung & Lee[2]	Unclear lesion boundaries. Clear lesion boundaries could not be discerned to accurately trace the lesion location. This case has bilateral lesions in the basal ganglia (caudate nucleus and putamen), central pons, and several cortical regions (insula and precentral frontal gyri).
Krauss & Jankovic[3]	Patient 3 of 3 presented. Unclear lesion boundaries. Clear lesion boundaries could not be discerned to accurately trace the lesion location. This case has cerebral atrophy with panventricular dilatation and widened convexity sulci and cerebellar fissures. Additionally, bilateral subcortical and periventricular leukoencephalopathic changes primarily of the frontal and right temporo-parietal white matter were noted.
Kjaer[4]	A large cyst displacing brain tissue. As mentioned, in line with previous lesion network mapping methods,[5] we excluded cases demonstrating large cysts distorting brain structure.
Lana-Peixoto & Teixeira[6]	Unclear lesion boundaries. Clear lesion boundaries could not be discerned to accurately trace the lesion location. This case has lesions in the subcortical white matter, corpus callosum, periventricular white matter, thalamus, basal ganglia, and brainstem.
Martino[7]	Poor image quality. Clear lesion boundaries could not be discerned to accurately trace the lesion location.
McAbee[8]	Unclear lesion boundaries. Clear lesion boundaries could not be discerned to accurately trace the lesion location. This case has multiple cystic areas in the left gyrus rectus and superior frontal gyrus.
Nociti[9]	Unclear lesion boundaries. Clear lesion boundaries could not be discerned to accurately trace the lesion location. This case has white matter lesions of both cerebral hemispheres, right cerebral peduncle, middle cerebellar peduncles, and both thalami.
Peterson[10]	Case 1 of 3 presented. The authors proposed genetic diathesis for the tics and obsessive-compulsive behaviors presented in this case. As previously mentioned, our inclusion of cases was guided by the author's judgment in the original published report.
Ranjan[11]	Unclear lesion boundaries. Clear lesion boundaries could not be discerned to accurately trace the lesion location. Additionally, this case has lesions across several regions of grey and white matter. This case has lesions in the corpus callosum, right putamen, dorsal aspect of the mesencephalon and over the high convexity at the grey/white matter interface.
Topçuoğlu[12]	Unclear lesion boundaries. Clear lesion boundaries could not be discerned to accurately trace the lesion location. This case has patchy ischemic regions in the basal ganglia, periventricular white matter, and centrum semiovale. Additionally, nonspecific ischemic regions in the right pons and left medulla oblongata were noted.

Supplementary Table 1 details the reasons for excluding cases of lesion-induced tics that were included by Ganos *et al.*[13].

Supplementary Table 2 Consistent regions of abnormality in Tourette syndrome

Contrast		Location	MNI			Volume (mm^3)	ALE Value	Convergence *n* (%)
			x	y	z			
**TS > HC**	1	L Thalamus/midbrain	-1.9	-12.7	-9.6	944	0.018	2 (33)
Studies in analysis = 6	2	L Midbrain	-3	-32.3	-14	400	0.011	2 (33)
	3	L Thalamus	-14	-30	9	160	0.010	1 (17)
	4	Midline Midbrain	0	-32.8	-5.4	136	0.010	1 (17)
**TS < HC**								
Studies in analysis = 6	1	R Frontal pole	13.6	63.6	10.6	304	0.013	2 (33)

Supplementary Table 2 presents significant clusters at $P < 0.001$ (uncorrected), cluster extent $100mm^3$. Center of gravity provided in Montreal Neurological Institute (MNI) xyz coordinates. *n* = number of studies contributing to clusters. R = right; L = left; TS = Tourette syndrome; HC = healthy control.

Supplementary Table 3 Demographic and clinical characteristics of participants: functional connectivity analyses

	**TS** (***n*** **= 21)**	**HC** (***n*** **= 25)**	
**Age,** ***Mean (SD)***	11.01 (2.54)	11.00 (2.23)	$t = 0.02$, $P = 0.99$
**Female sex,** ***n*** **(%)**	2 (9.5)	2 (8)	$P = 1.00$
**OCD,** ***n*** **(%)**	3 (14.3)	0 (0)	NA
**ADHD,** ***n*** **(%)**	12 (57.1)	0 (0)	NA
**Imaging site,** ***n*** **(%)**			$L\chi^2(2) = 0.22$, $P = 0.90$
RUBIC	13 (61.9)	14 (56)	
CBIC	7 (33.3)	10 (40)	
CUNY	1 (4.8)	1 (4)	
[a]**Medication used for behavior,** ***n*** **(%)**			NA
Stimulant for ADHD	2 (9.5)	0 (0)	
Non-stimulant for ADHD	3 (14.3)	0 (0)	
SSRI	2 (9.5)	0 (0)	

TS = Tourette syndrome; HC = healthy control; SD = standard deviation; OCD = obsessive compulsive disorder; ADHD = attention-deficit/hyperactivity disorder; RUBIC = Rutgers University Brain Imaging Center; CBIC = Citigroup Biomedical Imaging Center; CUNY = CUNY Advanced Science Research Center; SSRI = selective serotonin reuptake inhibitors. All clinical diagnoses were established using the Kiddie Schedule for Affective Disorders and Schizophrenia (K-SADS).[14] Expected values were not > 5 for the required number of cells for Chi-square Tests of Independence assessing differences in sex and imaging site between patients with Tourette syndrome and healthy controls. Accordingly, Fisher's Exact Test[15] and the Likelihood Ratio ($L\chi^2$)[16] were used to assess the significance of differences in sex and imaging site between groups, respectively. [a] = Some patients with Tourette syndrome were taking multiple medications, with five patients in total taking medication.

Supplementary Table 4 Regions of interest for the control seed-based analyses

	Study	Neurological disorder	ROI brain region
1	Corp[17]	Cervical dystonia	Bilateral cerebellum, somatosensory cortex
2	Joutsa[5]	Parkinsonism	Bilateral claustrum
3	Joutsa[18]	Holmes tremor	Cerebellar vermis, flocculonodular, pontomedullary junction, midbrain

To assess whether connectivity from our conjunction networks was selectively abnormal in patients with Tourette syndrome, we replicated the seed-based analysis in the 'sensitive and specific' LNM networks identified in the control datasets involving other movement disorders (cervical dystonia, parkinsonism, and Holmes tremor). Connectivity did not significantly differ between patients and controls from any control regions of interest (ROIs) derived from these other movement disorders.

Supplementary Table 5 Case characteristics of lesions causing tics

Case No/Ref	Year	Age at lesion identification (y)/Age at tic onset (y)/Sex	Lesion etiology	Lesion location	Tic symptom latency[a] (months)	Tic symptom	Suppressibility/ premonitory urge	Comorbid movement disorder	Neuropsychiatric comorbidity
1) Alioğlu[19]	2004	12/12/M	Carbon monoxide intoxication	BG (CN/putamen)	0.4	L arm & leg movements, smiling, bilateral shoulder shrug, arm jerk, foot dorsiflexion; speech arrest, throat clearing, humming, lip smacking	NA/NA	None	NA
2) Berthier[20]	2003	12/NA/M	Hypoxic-ischemic damage	R midbrain, thalamus, BG (L CN/putamen), internal capsule	NA	Eye blinking, grimacing, R facial tic, head jerk, body rocking; throat clearing, whistling, repetitive speech, echo/coprolalia	Yes/NA	Dystonia, fine and gross motor deficits	ASD, restrictive social interaction, unusual preoccupations, fixed interest, rigid insistence, compulsive rituals (checking, smelling, touching), inattentiveness, impulsivity, violent outbursts, self-injurious behaviors
3) Boca[21]	2016	16/16/M	Acute necrotizing encephalopathy	BG (CN, putamen, GP), thalami, CS	NA	Motor/vocal tics, grinning; palilalia	NA/NA	Parkinsonism	ADHD, OCD, over-fast writing script
4) Cheng[22]	2019	48/48/F	Tumor associated with Lhermitte-Duclos disease	L cerebellum	NA	L facial tics	NA/NA	None	Occipital headache, dizziness

5) Criscuolo[23]	2017	NA/47/M	TBI	GP	NA	Arm abduction, shoulder lifting, hip twisting, eye blinking, forehead wrinkling, lower jaw lateral deviation	Yes/Yes	None	NA
6) Demirkol[24]	1999	17/NA/M	NA	GP	NA	Head jerk, shoulder shrugging, cheek/lip/tongue touching, spitting into palms; mouth smacking, explosive laughter, sniffing, echo/coprolalia	NA/NA	Coordination difficulties and associated gait disorder	ADHD, OCD, stuttering; antisocial, aggressive, self-injurious and impulsive behaviors
7) de Souza[25]	2020	28/35/M	Head trauma from nail gun	L parietal lobe	84	Episodic head turning; repetitive vocalizations ("me" & "boo"), coprolalia	Yes/Yes	None	NA
8) Edwards[26]	2004	27/27/F[b]	NA	BG (R CN/LN)	NA	Facial grimacing, eyebrow raising, shoulder elevation	Yes/Yes	Stereotypies of right hand/foot	Memory deficits, anxiety
9) Gomis[27]	2008	71/71/M	Ischemic stroke	L CN	0.75	Involuntary ("a") vocalization	Yes/Yes	Gait disturbance	None
10) Ko[28]	2004	36/27/F	Carbon monoxide intoxication	GP	1	Facial contortion with mouth opening, lateral jaw movement, neck twist, shoulder shrug	Yes/Yes	None	NA
11) Kwak & Jankovic[29]	2002	NA/8.5/M[c]	Hemorrhagic stroke	BG (R putamen/CN)	6	Eye blinking, head jerk, shoulder shrug, compulsive scratching, rapid clonic head nodding, neck thrusting; blowing air onto palms	Yes/Yes	Dystonia	ADHD, OCD, repetitive scratching

12) Liu[30]	1999	8/8/F	Germinoma	L BG	NA	L shoulder tic	NA/NA	None	Deficits in memory and attention, reduced language functioning, distractibility, compulsions (saying "nice tie" whenever seeing someone with a tie)
13) Lua[31]	2012	57/57/F	Vestibular schwannoma	L cerebellopontine angle/brainstem	NA	L facial tics	NA/NA	None	Paresthesia, hearing loss
14) Luat[32]	2009	12/11/M	Oligodendroglioma	R temporal lobe, extending into the BG, hypothalamus	NA	Head jerk, eye blinking; repeated coughing, throat clearing	NA/NA	None	Inattention, disruptive behavior, hyperactivity, severe obsessive-compulsive behavior, generalized anxiety, seizures
15) Majumdar & Appleton[33]	2002	7.5/8/F	TBI	BG (L putamen/GP/CN), internal capsule	15	Eye blinking, shoulder/arm jerk, trunk flexation, echopraxia; grunts, clicking of tongue, echo/coprolalia	Yes/NA	Ataxia	Attention and memory deficits, behavioral disinhibition
16) Nardocci[34]	1994	17/11/M	NA	GP	NA	Eye blinking, forced staring, head jerk, fluttering of hands, repetitive hitting of knee with fist, touching forehead with licked finger, facial grimacing, forced smiling; palilalia	NA/NA	Dystonia, stereotypies	ADHD, Hallervorden-Spatz Disease, social maladaptation, obsessive compulsive behaviors (ruminating thoughts, repetitive touching of objects/body parts), apathy, irritability

17) Northam & Singer[35]	1991	6/6/F	Herpes encephalitis	R temporal lobe	0.5	Eye blinking, facial grimacing, head twitch, shoulder shrug, eye rolling, facial contortions, jumping, touching objects and body parts, copropraxia; grunting, sniffing, snorting	Yes/NA	Overflow movements (potential Dystonia)	Marked personality changes, emotional lability, severe anxiety, hyperactivity
18) Pan & Zhang[36]	2018	52/52/M	Histiocytic sarcoma	L frontal lobe	NA	Head motor tics	NA/NA	None	Expressive aphasia
19) Yochelson & David[37]	2000	16/16/M	Arteriovenous malformation	L frontal lobe	0.07	Head turning; vocalization ("yeah")	Yes/Yes	None	NA

Italicized case numbers and author names represent cases that were not included by Ganos *et al.*[13]; [a] = duration between lesioning event and tic onset; [b] = case 1 of 4 reported; [c] = patient 1 of 2 reported; NA = not available; ADHD = attention-deficit/hyperactivity disorder; OCD = obsessive compulsive disorder; ASD = autism spectrum disorder; TBI = traumatic brain injury; BG = basal ganglia; CN = caudate nucleus; GP = globus pallidus; LN = lentiform nucleus; CS = centrum semiovale; R = right; L = left.

Supplementary Table 6 Participant characteristics and neuroimaging results of studies included in the ALE and CNM analyses

Study	Number (female)		Mean age (years), SD		Comorbidity (%)	YGTSS (x̄ ± SD)	Method	Neuroimaging results
	TS	HC	TS	HC				
1) Draganski[38]	40 (10)	40 (15)	32.4 ± 11	34.4 ± 9	ADHD (47.5); OCD (35)	28.7 ± 7.4	VBM	TS patients showed lower GMV in the rostral cingulate, medial orbitofrontal, ventrolateral prefrontal cortex, operculum; greater GMV in the bilateral putamen
2) Garraux[39]	31 (6)	31 (8)	32 ± 10.5	32 ± 11	ADHD (32.3); OCD (38.7)	31.6 ± 11.8	VBM	TS patients showed greater GMV in the left midbrain
3) Greene[40]	103 (22)	103 (22)	11.9 ± 2.1	11.9 ± 2.1	ADHD (55)[a]; OCD (47)[a]	18.1 ± 8.3	VBM	TS patients showed greater GMV in the left pulvinar nucleus (thalamus), ventral midbrain, bilateral hypothalamus; lower WMV in the orbital and medial prefrontal cortex
4) Liu[41]	21 (1)	20 (3)	7.90 ± 1.95	8.05 ± 2.30	None	41.71 ± 12.46	VBM	TS patients showed lower GMV in the left superior temporal gyrus; greater GMV in the bilateral precentral gyrus; lower WMV in the right precuneus, right precentral gyrus, right frontal lobe, right postcentral gyrus, left lingual gyrus, left temporal occipital fusiform cortex
5) Ludolph[42]	14 (0)	15 (0)	12.5	13.4	NA	NA	VBM	TS patients showed greater GMV in the bilateral ventral putamen; lower GMV in the bilateral hippocampus
6) Müller-Vahl[43]	19 (0)	20 (0)	30.4	31.7	None	28.8	VBM	TS patients showed lower GMV in the left caudate nucleus, ACC, primary sensorimotor areas, right middle and medial frontal gyri; lower volume of the WM below the right inferior frontal gyrus, anterior corpus callosum, left superior frontal gyrus; greater WMV in the left middle frontal gyrus and primary sensorimotor region
7) Wittfoth[44]	29 (0)	24 (0)	30.7 ± 9	30.6 ± 10.9	OCD (58.6); ADHD (13.8); OCD+ADHD (27.6)	35.7 ± 17.6	VBM	TS patients showed lower GMV in the left inferior frontal gyrus

TS = Tourette syndrome; HC = healthy control; SD = standard deviation; ADHD = attention-deficit/hyperactivity disorder; OCD = obsessive compulsive disorder; [a] = not available for all participants; GMV = grey matter volume; WMV = white matter volume; ACC = anterior cingulate cortex; YGTSS = Yale Global Tic Severity Scale; VBM = voxel-based morphometry.

Supplementary Table 7 Coordinates used in the ALE and CNM analyses

	MNI		
**Study**	**x**	**y**	**z**
Draganski[38]	-53	35	-6
	45	38	-12
	0	33	-26
	-39	-3	7
	58	-13	16
	7	44	1
	-27	-10	3
	27	-3	9
Garraux[39]	-7	-29	-14
	-2	-13	-11
	-7	-17	-4
Greene[40]	-13.5	31.5	-22.5
	-12.4	9.5	-16.5
	-15	22.5	-21
	-19.5	51	9
	-19.5	43.5	-12
	-13.5	36	15
	-15	55.5	9
	-16.5	46.5	3
	-18	51	-7.5
	-27	55.5	-10.5
	-22.5	36	21
	7.5	49.5	-19.5
	18	42	3
	16.5	55.5	-15
	15	63	7.5
	21	43.5	-7.5
	7.5	58.5	-13.5
	30	49.5	-12
	13.5	42	-19.5
	-13.5	-30	9
	-15	-28.5	-4.5
	0	-33	-4.5
	0	-34.5	-13.5
	9	-3	-16.5
	-1.5	-6	-7.5
	0	-15	-10.5
Liu[41]	-58	-36	14
	-12	-28	72
	18	-24	68

	16	-52	50
	14	-24	78
	-40	-56	-12
	12	64	14
	16	-38	64
	10	-34	72
	-20	-56	2
Ludolph[42]	25	13	-5
	-20	17	-6
	27	-19	-17
	-20	-19	-21
Müller-Vahl[43]	-55	-8	20
	38	-20	47
	9	22	31
	-15	14	13
	-10	11	3
	-36	12	56
	-45	-18	36
	-52	-15	40
	44	6	57
	59	-13	38
	45	-19	38
	41	-26	58
	10	29	48
	-9	12	39
	44	3	25
	-16	15	54
	-25	-20	70
	7	34	10
	-32	-77	-12
	-54	-10	20
	-26	-12	53
	-19	-11	62
	-45	-18	38
	27	-46	6
	49	10	11
Wittfoth[44]	36	38	3

Supplementary Table 7 presents the coordinates used in the ALE and CNM analyses, provided in MNI xyz.

References

1. Dale RC, Church AJ, Heyman I. Striatal encephalitis after varicella zoster infection complicated by Tourettism. *Mov Disord*. 2003;18(12):1554-1556. doi:10.1002/mds.10610
2. Jung NY, Lee JH. Secondary tics after osmotic demyelination syndrome involving both the striatum and the cerebral cortex. *J Clin Neurosci*. 2012;19(1):179-180. doi:10.1016/j.jocn.2011.04.022
3. Krauss JK, Jankovic J. Tics secondary to craniocerebral trauma. *Mov Disord*. 1997;12(5):776-782. doi:10.1002/mds.870120527
4. Kjaer M, Boris P, Gadegaard Hansen L. Abnormal CT scan in a patient with Gilles de la Tourette syndrome. *Neuroradiology*. 1986;28(4):362-363. doi:10.1007/BF00333447
5. Joutsa J, Horn A, Hsu J, Fox MD. Localizing parkinsonism based on focal brain lesions. *Brain*. 2018;141(8):2445-2456. doi:10.1093/brain/awy161
6. Lana-Peixoto MA, Teixeira AL, The Brazilian Committee for Treatment and Research in Multiple Sclerosis (BCTRIMS). Simple phonic tic in multiple sclerosis. *Mult Scler J*. 2002;8(6):510-511. doi:10.1191/1352458502ms829oa
7. Martino D, Church A, Giovannoni G. Are antibasal ganglia antibodies important, and clinically useful? *Pract Neurol*. 2007;7(1):32-41.
8. McAbee GN, Wark JE, Manning A. Tourette syndrome associated with unilateral cystic changes in the gyrus rectus. *Pediatr Neurol*. 1999;20(4):322-324. doi:10.1016/S0887-8994(98)00159-3
9. Nociti V, Fasano A, Bentivoglio AR, et al. Tourettism in Multiple Sclerosis: A case report. *J Neurol Sci*. 2009;287(1-2):288-290. doi:10.1016/j.jns.2009.07.009
10. Peterson BS, Bronen RA, Duncan CC. Three cases of symptom change in Tourette's syndrome and obsessive-compulsive disorder associated with paediatric cerebral malignancies. *J Neurol Neurosurg Psychiatry*. 1996;61(5):497-505. doi:10.1136/jnnp.61.5.497
11. Ranjan N, Nair KPS, Romanoski C, Singh R, Venketswara G. Tics after traumatic brain injury. *Brain Inj*. 2011;25(6):629-633. doi:10.3109/02699052.2011.572944
12. Topçuoğlu V, Arman A, Yazgan C, Arman A, Gönentür A, Bicer D. Tourettism in a 73 Year Old Man After Coronary Artery Bypass Surgery. Published online 2009.
13. Ganos C, Al-Fatly B, Fischer JF, et al. A neural network for tics: insights from causal brain lesions and deep brain stimulation. *Brain*. Published online January 13, 2022:awac009. doi:10.1093/brain/awac009
14. Kaufman J, Birmaher B, Brent D, et al. Schedule for Affective Disorders and Schizophrenia for School-Age Children-Present and Lifetime Version (K-SADS-PL): Initial Reliability and Validity Data. *J Am Acad Child Adolesc Psychiatry*. 1997;36(7):980-988. doi:10.1097/00004583-199707000-00021
15. Fisher RA. On the Interpretation of χ 2 from Contingency Tables, and the Calculation of P. *J R Stat Soc*. 1922;85(1):87. doi:10.2307/2340521
16. Field A. *Discovering Statistics Using IBM SPSS Statistics*. 5th edition. SAGE Publications; 2017.
17. Corp DT, Joutsa J, Darby RR, et al. Network localization of cervical dystonia based on causal brain lesions. *Brain*. 2019;142(6):1660-1674. doi:10.1093/brain/awz112
18. Joutsa J, Shih LC, Fox MD. Mapping holmes tremor circuit using the human brain connectome. *Ann Neurol*. 2019;86(6):812-820. doi:10.1002/ana.25618
19. Alioğlu Z, Boz C, Sari A, Aynaci M. Transient tic disorder following carbon monoxide poisoning. *J Neuroradiol*. 2004;31(3):231-233. doi:10.1016/S0150-9861(04)96999-4
20. Berthier ML, Kulisevsky J, Asenjo B, Aparicio J, Lara D. Comorbid Asperger and Tourette syndromes with localized mesencephalic, infrathalamic, thalamic, and striatal damage. *Dev Med Child Neurol*. 2003;45(03). doi:10.1017/S0012162203000392

21. Boca M, Lloyd K, Likeman M, Jardine P, Whone A. Basal ganglia necrosis: a 'best-fit' approach. *Pract Neurol*. 2016;16(6):458-461. doi:10.1136/practneurol-2016-001410
22. Cheng CS, Ou CH, Chen JS, Lui CC, Yeh LR. Lhermitte-Duclos disease: A case report with radiologic-pathologic correlation. *Radiol Case Rep*. 2019;14(6):734-739. doi:10.1016/j.radcr.2019.03.020
23. Criscuolo C, D'Amico A, Peluso S, Popolizio T, Pellecchia MT. Adult-onset pure tic disorder after post-traumatic hypoxic lesions of the globus pallidus. *Parkinsonism Relat Disord*. 2017;34:75-76. doi:10.1016/j.parkreldis.2016.11.007
24. Demirkol A, Erdem H, Inan L, Yigit A, Güney M. Bilateral globus pallidus lesions in a patient with Tourette syndrome and related disorders. *Biol Psychiatry*. 1999;46(6):863-867. doi:10.1016/S0006-3223(99)00087-6
25. de Souza A. New-onset tic disorder following circumscribed brain injury. *J Clin Neurosci*. 2020;75:234-239. doi:10.1016/j.jocn.2020.03.009
26. Edwards MJ, Dale RC, Church AJ, et al. Adult-onset tic disorder, motor stereotypies, and behavioural disturbance associated with antibasal ganglia antibodies. *Mov Disord*. 2004;19(10):1190-1196. doi:10.1002/mds.20126
27. Gomis M, Puente V, Pont-Sunyer C, Oliveras C, Roquer J. Adult onset simple phonic tic after caudate stroke. *Mov Disord*. 2008;23(5):765-766. doi:10.1002/mds.21955
28. Ko SB, Ahn TB, Kim JM, Kim Y, Jeon BS. A Case of Adult Onset Tic Disorder Following Carbon Monoxide Intoxication. *Can J Neurol Sci J Can Sci Neurol*. 2004;31(2):268-270. doi:10.1017/S0317167100053944
29. Kwak CH, Jankovic J. Tourettism and dystonia after subcortical stroke. *Mov Disord*. 2002;17(4):821-825. doi:10.1002/mds.10207
30. Liu E, Robertson RL, du Plessis A, Pomeroy SL. Basal ganglia germinoma with progressive cerebral hemiatrophy. *Pediatr Neurol*. 1999;20(4):312-314. doi:10.1016/S0887-8994(98)00161-1
31. Lua BK, Lieu AS, Hwang SL. Breast carcinoma metastasized to vestibular schwannoma: A rare case of tumor-to-tumor metastasis and literature review. *Kaohsiung J Med Sci*. 2012;28(7):397-399. doi:10.1016/j.kjms.2012.02.007
32. Luat AF, Behen ME, Juhász C, Sood S, Chugani HT. Secondary Tics or Tourettism Associated With a Brain Tumor. *Pediatr Neurol*. 2009;41(6):457-460. doi:10.1016/j.pediatrneurol.2009.07.009
33. Majumdar A, Appleton RE. Delayed and severe but transient Tourette syndrome after head injury. *Pediatr Neurol*. 2002;27(4):314-317. doi:10.1016/S0887-8994(02)00446-0
34. Nardocci N, Rumi V, Combi ML, Angelini L, Mirabile D, Bruzzone MG. Complex tics, stereotypies, and compulsive behavior as clinical presentation of a juvenile progressive dystonia suggestive of hallervorden-spatz disease. *Mov Disord*. 1994;9(3):369-371. doi:10.1002/mds.870090322
35. Northam RS, Singer HS. Postencephalitic acquired Tourette-like syndrome in a child. *Neurology*. 1991;41(4):592-592. doi:10.1212/WNL.41.4.592
36. Pan Y, Zhang Y. Simultaneous Brain and Lung Histiocytic Sarcoma Revealed on 18F-FDG PET/CT. *Clin Nucl Med*. 2018;43(1):65-67. doi:10.1097/RLU.0000000000001908
37. Yochelson MR, David RG. New-Onset Tic Disorder Following Acute Hemorrhage of an Arteriovenous Malformation. *J Child Neurol*. 2000;15(11):769-771. doi:10.1177/088307380001501114
38. Draganski B, Martino D, Cavanna AE, et al. Multispectral brain morphometry in Tourette syndrome persisting into adulthood. *Brain*. 2010;133(12):3661-3675. doi:10.1093/brain/awq300

39. Garraux G, Goldfine A, Bohlhalter S, Lerner A, Hanakawa T, Hallett M. Increased midbrain gray matter in Tourette's syndrome. *Ann Neurol*. 2006;59(2):381-385. doi:10.1002/ana.20765
40. Greene DJ, Williams III AC, Koller JM, Schlaggar BL, Black KJ, and The Tourette Association of America Neuroimaging Consortium. Brain structure in pediatric Tourette syndrome. *Mol Psychiatry*. 2017;22(7):972-980. doi:10.1038/mp.2016.194
41. Liu Y, Miao W, Wang J, et al. Structural Abnormalities in Early Tourette Syndrome Children: A Combined Voxel-Based Morphometry and Tract-Based Spatial Statistics Study. Huang H, ed. *PLoS ONE*. 2013;8(9):e76105. doi:10.1371/journal.pone.0076105
42. Ludolph AG, Juengling FD, Libal G, Ludolph AC, Fegert JM, Kassubek J. Grey-matter abnormalities in boys with Tourette syndrome: magnetic resonance imaging study using optimised voxel-based morphometry. *Br J Psychiatry*. 2006;188(5):484-485. doi:10.1192/bjp.bp.105.008813
43. Müller-Vahl KR, Kaufmann J, Grosskreutz J, Dengler R, Emrich HM, Peschel T. Prefrontal and anterior cingulate cortex abnormalities in Tourette Syndrome: evidence from voxel-based morphometry and magnetization transfer imaging. *BMC Neurosci*. 2009;10(1):47. doi:10.1186/1471-2202-10-47
44. Wittfoth M, Bornmann S, Peschel T, et al. Lateral frontal cortex volume reduction in Tourette syndrome revealed by VBM. *BMC Neurosci*. 2012;13(1):17. doi:10.1186/1471-2202-13-17
